# Loss of miR-101-3p in melanoma stabilizes genomic integrity, leading to cell death prevention

**DOI:** 10.1186/s11658-024-00552-2

**Published:** 2024-03-02

**Authors:** Lisa Lämmerhirt, Melanie Kappelmann-Fenzl, Stefan Fischer, Paula Meier, Sebastian Staebler, Silke Kuphal, Anja-Katrin Bosserhoff

**Affiliations:** 1 Institute of Biochemistry, Friedrich-Alexander University Erlangen-Nürnberg (FAU), Fahrstraße 17, 91054 Erlangen, Germany; 2 Faculty of Computer Science, Deggendorf Institute of Technology, Dieter-Görlitz-Platz 1, 94469 Deggendorf, Germany; 3 Julius-Maximilians-University Würzburg (JMU), Sanderring 2, 97070 Würzburg, Germany

**Keywords:** Melanoma, miRNA, DNA damage, Apoptosis, RNA-seq, Genomic integrity, miR-101-3p

## Abstract

Malignant melanoma remains the most lethal form of skin cancer, exhibiting poor prognosis after forming distant metastasis. Owing to their potential tumor-suppressive properties by regulating oncogenes and tumor suppressor genes, microRNAs are important player in melanoma development and progression. We defined the loss of miR-101-3p expression in melanoma cells compared with melanocytes and melanoblast-related cells as an early event in tumor development and aimed to understand the tumor suppressive role of miR-101-3p and its regulation of important cellular processes. Reexpression of miR-101-3p resulted in inhibition of proliferation, increase in DNA damage, and induction of apoptosis. We further determined the nuclear structure protein Lamin B1, which influences nuclear processes and heterochromatin structure, ATRX, CASP3, and PARP as an important direct target of miR-101-3p. RNA sequencing and differential gene expression analysis after miR-101-3p reexpression supported our findings and the importance of loss of mir-101-3p for melanoma progression. The validated functional effects are related to genomic instability, as recent studies suggest miRNAs plays a key role in mediating this cellular process. Therefore, we concluded that miR-101-3p reexpression increases the genomic instability, leading to irreversible DNA damage, which leads to apoptosis induction. Our findings suggest that the loss of miR-101-3p in melanoma serves as an early event in melanoma progression by influencing the genomic integrity to maintain the increased bioenergetic demand.

**Supplementary Information:**

The online version contains supplementary material available at 10.1186/s11658-024-00552-2.

## Introduction

Malignant melanoma stands as one of the most lethal forms of skin cancer globally, exhibiting a consistently rising incidence [1]. If detected early, melanoma patients can be cured by complete resection of the primary tumor. However, as melanoma is an early metastasizing tumor entity, it results in poor prognosis and ends fatally in 65% of patients after forming distant metastasis [1, 2]. Because of steadily evolving resistances and alterations, treatment options are still challenging and limited [3]. Genomic instability, as a hallmark of cancer, contributes to tumor heterogeneity caused by defects in DNA damage surveillance mechanisms, mitotic checkpoints, and the DNA repair machinery [4]. Furthermore, genomic instability, known to be an early event in driving cells toward malignancy, has been extensively studied for its epigenetic role in cancer evolution [5–7]. MicroRNAs (miRNAs), which modulate gene expression and thereby pathway activities, represent another field to study the instability of the genome and carcinogenesis [4]. Discovered in 1993, miRNAs, as short single-stranded noncoding RNAs, play a crucial role in the epigenome and remain a significant field of investigation [8, 9]. Moreover, miRNAs are involved in regulating posttranscriptional gene expression by binding complementary with their 3′-untranslated region (UTR) seed sequence on the respective mRNA, leading to translational repression [10]. Thus, microRNAs are key players in various biological processes such as cellular development, differentiation, and proliferation [11]. They can act as either oncogenes or tumor suppressors by targeting genes involved in pro-oncogenic or anti-oncogenic pathways [12]. The functional effects of miRNAs as tumor suppressors are variable, influencing biological processes such as proliferation, migration, immune checkpoints, metabolism, and DNA damage in different types of cancer [11, 13–16]. Our own studies support this variability, demonstrating that the reexpression of miR-488-5p in melanoma cells induces apoptosis [17] and miR-622 acts as a tumor suppressor by targeting Kirsten rat sarcoma (KRAS) [18]. Additionally, we have already been able to show that miR-125b reexpression leads to reduced proliferation and migration and targets c-Jun, a main regulator of tumor progression [19]. Recent studies indicate that miRNAs are involved in mediating mitotic regulation and DNA damage response, underpinning their role in affecting genomic instability [20–22]. The exact mechanisms by which miRNAs can cause genomic instability have only been revealed recently [4]. The fact that microRNAs regulate expression of up to 30% of human protein-coding genes implies a broad spectrum of possibilities in finding some new therapy approaches using miRNAs [23]. The important role of miR-101-3p has been described in some tumor entities. For instance, miR-101-3p promotes cell apoptosis in oral cancer [24], induces autophagy in endometrial carcinoma cells [25], and inhibits the progression of lung squamous cell carcinoma cell lines [26]. In this study, we addressed the functional and molecular role of miR-101-3p in melanoma, specifically examining its impact on crucial cellular processes and structures such as cell division and DNA damage, leading to genomic instability and apoptosis.

## Materials and methods

### Cultivation of melanocytes and dedifferentiation into melanoblast-related cells (MBrCs)

The cultivation of normal human epidermal melanocytes (NHEMs) was performed as described previously [27]. For experiments, NHEMs from PromoCell (Heidelberg, Germany) or Lonza (Basel, Switzerland) were used. NHEMs are derived from human neonatal foreskin tissue donors with low melanin levels and were grown either in a melanocyte serum-free M2 medium without phorbol myristate acetate (PMA) (PromoCell) or medium with PMA (Lonza) at 37 °C and 5% CO_2_.

For dedifferentiation into MBrCs, NHEMs were grown for three passages and subsequently cultivated in a melanoblast growth medium for 14 days. The medium consisted of the following components: MCBD 153 medium (Sigma-Aldrich) containing 8% chelated fetal bovine serum (FBS), 2% normal FBS (PAA Laboratories, Pasching, Austria), 2 mM glutamine, 1.66 ng/ml cholera toxin B, 10 ng/ml SCF (Sigma-Aldrich), 100 nM endothelin-3, and 2.5 ng/ml bFGF. Chelated FBS was prepared by mixing 1.2 g of Chelex-100 (Sigma-Aldrich) per 40 ml of FBS for 1.5 h at 4 °C with gentle stirring. The dedifferentiation procedure of melanocytes to MBrCs is published by Cook et al. and Larribère et al. [28–30].

### Melanoma cell culture

The primary melanoma cell line MEL-JUSO was cultivated in Roswell Park Memorial Institute (RPMI) culture medium supplemented with 10% fetal bovine serum (FBS), penicillin (400 U/ml), streptomycin (50 µg/ml), and 0.2% sodium bicarbonate (all from Sigma-Aldrich). The cell lines derived from melanoma metastases, SK-MEL-28 and MV3, were cultivated in low-glucose Dulbecco’s modified Eagle’s medium (DMEM) or high-glucose DMEM with 10% FBS, penicillin (400 U/ml), and streptomycin (50 µg/ml) (Sigma-Aldrich). The melanoma cells were incubated in a humidified atmosphere containing 8% at 37 °C in T75 culture flasks (Corning Incorporated, NY, USA).

The fluorescence ubiquitination-based cell cycle indicator (FUCCI) system was stably integrated into MV3 by lentiviral transduction of plasmid pBOB-EF1-FastFUCCI-Puro, as previously described [31, 32]. Expression of the fluorescence coupled cell cycle proteins Chromatin licensing and DNA replication factor 1 (Cdt1) (red) or geminin (green) indicate either G1 or S/G2/M state of the cells. During the transition from G1 to S phase, both proteins are present and merge, producing a yellow fluorescence signal. The resulting cell line MV3 FUCCI was cultivated like the parental cell line MV3. To avoid the loss of the reporter, 4 µg/ml puromycin (Sigma Aldrich) was added every second week for selection.

### Transient transfection of microRNAs

NHEM and human melanoma cell lines MEL-JUSO, SK-MEL-28, and MV3 were transfected with a microRNA mimic of miR-101-3p (Syn-hsa-miR-101-3p miScript miRNA mimic GeneGlobe ID-MSY0000099, cat. no. 219600/) and siCtrl (AllStars Neg. Control siRNA, cat. no. ID:1027281), respectively (Qiagen, Hilden, Germany), using the Lipofectamine RNAiMAX reagent (Life Technologies, Darmstadt, Germany) according to the manufacturer's instruction as described previously [33]. For the transfection, 150,000–200,000 cells were seeded into each well of a six-well plate. Each transfection batch contained 50 pmol mimic from a 20 µM stock solution. A transfection mixture for a six-well was prepared in 500 µl total volume of transfection medium without Phenol Red and without FBS. The transfection was performed for the respective functional assays.

### microRNA expression analysis

Isolation of total cellular miRNA from cultured human melanoma cell lines and reverse transcription (RT) into miRcDNA was performed as described previously [34] or using the isolation kit miRNeasy Tissue/Cells Advanced Micro Kit (Qiagen) according to the manufacturers’ specifications. For expression analysis of miR-101-3p in different melanoma cell lines, qRT-PCR was used as described previously [35] using miScript-System with miR-101-3p and U6 as control according to the manufacturers’ protocol (Qiagen). Additionally, for the expression analysis of miR-101-3p after miR-101-3p reexpression, qRT-PCR was used, using the miRCURY-System of the miRCURY LNA SYBR Green PCR Kit with the hsa-miR-101-3p miRCURY LNA miRNA PCR assay and U6 SNRNA miRCURY LNA miRNA PCR assay (Qiagen). For each qRT-PCR, 500 ng of miRNA was reversed-transcribed into micDNA on the basis of the manufacturers’ protocol.

### Immunofluorescence staining

For the analyses of the nuclear proteins PML and LMNB1, immunofluorescence staining was performed as described previously [36]. Here, the melanoma cells were treated with miR-101-3p mimic for 72 h, then harvested, counted, and 25,000 or 30,000 cells were seeded for each treatment onto round 18-mm cover slips (Carl-Roth, Karlsruhe, Germany) in 12-well culture plates (Corning Incorporated). Before staining, the adherent cells were fixed and permeabilized with ice-cold methanol for 5 min and blocked with 10% bovine serum albumin (BSA) in PBS. The cover slips were incubated overnight with the primary 1:200 rabbit anti-PML antibody (Santa Cruz Biotech, Heidelberg, Germany) or 1:1000 rabbit anti-LMNB1 antibody (Abcam, Cambridge, UK) at 4 °C. On the following day, the cover slips were incubated for 1 h with the secondary Cy3 antibody (1:500, Biozol, Eching, Germany). Thereafter, the cover slips were incubated in 4′,6-diamidino-2-phenylindole (DAPI) solution (1:10,000) (Merck KGaA, Darmstadt, Germany) in 1% BSA/PBS for 30 min. As mounting medium, Aqua-Poly/Mount (US Headquarters Polysciences, Warrington, PA, USA) was used. Immunofluorescence staining was analyzed with an IX83 microscope with Olympus CellSens Dimension software (version 2.3; Olympus, Hamburg, Germany).

### Western blot analysis

Using radioimmunoprecipitation assay (RIPA) buffer, the cell pellets were lysed and the total protein concentration was determined using the Pierce BCA Protein Assay Kit (Thermo Fisher Scientific Inc., Rockford, IL, USA) as previously described [37]. For western blot analysis, 30 µg of total protein lysate of each sample was separated on a 12.75% sodium dodecyl sulfate (SDS) polyacrylamide gel for electrophoresis. For detection of the proteins separated by SDS-PAGE with specific antibodies, the proteins were transferred to a polyvinylidene fluoride (PVDF) membrane. The following primary antibodies were used for analyses: mouse anti-beta-actin (1:5000, Sigma Aldrich), rabbit anti-PARP (1:1000, Cell Signaling Technology, Danvers, MA, USA), rabbit anti-LMNB1 (1:1000, Abcam), and rabbit anti-γH2AX (1:1000, Cell Signaling Technology). By combining specific primary antibodies diluted as described and the corresponding labeled secondary antibody, the individual proteins were detected. The following secondary horseradish peroxidase-coupled antibodies were used: 1:2000, anti-rabbit horseradish peroxidase (HRP) or anti-mouse HRP (Cell Signaling Technology, Frankfurt, Germany). To detect the HRP-coupled secondary antibody, the Clarity™ Western ECL Substrate of the ECL Plus Western Blotting Detection Kit (GE Healthcare Life Science Europe GmbH, Freiburg, Germany) was added to the membrane. The resulting chemiluminescence was detected using an Intas Chemostar imager (Intas Science Imaging Instruments GmbH, Göttingen, Germany). Densitometric analysis of the signals was performed using LabImage software (Kapelan Bio Imaging GmbH, Leipzig, Germany).

### Clonogenic assay

The clonogenic assay was used for analysis of stem cell behavior and proliferation in an attachment-dependent manner of colony formation [38]. For this, 500 NHEMs or melanoma cells of the cell lines MEL-JUSO, SK-MEL-28, and MV3, transfected with miR-101-3p mimic or the respective control, were seeded into a well of a six-well plate. The cells were incubated for 7 days at 37 °C with 8% CO_2_ content. Subsequently, the cells were fixed and stained in the wells with 400 µl of a mixture of 6% glutaraldehyde (Sigma-Aldrich) and 0.36% Crystal Violet (Sigma-Aldrich) for 30 min. Afterwards, the cells were washed several times with tap water, until the water was no longer stained. After drying the six-well plates overnight at room temperature, the plates were scanned and then evaluated with Olympus CellSens Dimension software (version 2.3, Olympus).

### Analysis of cell proliferation via real-time cell analysis (RTCA)

Real-time cell proliferation was determined using the xCELLigence system (Roche, Mannheim, Germany) (“E-Plates”) as described earlier [39]. For melanoma cell lines (MEL-JUSO, SK-MEL-28, and MV3), 2000–3000 cells and for NHEMs 5000 cells transfected with miR-101-3p mimic and siCtrl, respectively, are shown in duplicates.

### Cell cycle analysis with FUCCI reporter

For cell cycle quantification, the FUCCI reporter cells, as described in Sect. 2.2, were used. For this, 50,000 cells were seeded per well of a six-well plate and transfected for 72 h with siCtrl or miR-101-3p mimic, respectively. MV3 FUCCI cells were analyzed and quantified using an Olympus IX83 microscope in the CY3 (533–559 nm) or GFP (450–490 nm) filter. For quantification, the Cell Counter plugin of Fiji ImageJ software (version 152n, see 6.17) was used. With the help of this software, the cells on the previously recorded fluorescence images were counted as red (G1), green (S/G2/M), or yellow (G1/S). The percentage of cells in the respective cell cycle phase was always set in relation to the total cell count.

### Cell cycle analysis with propidium iodide flow cytometry

To analyze the cell cycle of different melanoma cell lines treated with miR-101-3p mimic and siCtrl for 72 h, respectively, propidium iodide dye was used as nucleic acid intercalator. This assay was performed using flow cytometry as previously described [40] using a BD LSRFortessa™ flow cytometer in combination with BD FACSDiva™ software (version 8.0; BD Biosciences, San Jose, CA, USA).

### Analysis of apoptosis by flow cytometry

For detection and quantification of apoptotic cells, the Annexin V-FITC apoptosis kit (MyBioSource, San Diego, CA, USA) for flow cytometry was used according to the manufacturer’s instructions and performed as described elsewhere [41]. After transfection of the cells with miR-101-3p mimic or siCtrl, respectively, for 72 h, the cells were harvested following the protocols described above. Samples were analyzed using a BD LSRFortessa™ flow cytometer in combination with BD FACSDiva™ software (version 8.0; BD Biosciences, San Jose, CA, USA).

### Comet assay

For the detection of cellular DNA damage, a comet assay, also called a single-cell gel electrophoresis assay, was used. This method visualizes DNA damage, using a fluorescent dye. The Comet Assay Kit (Abcam) was used to perform the assay, and the required reagents were prepared according to the manufacturer’s instructions. For analysis, 150,000 cells (MV3) were transfected with miR-101-3p mimic in a six-well plate for 72 h. After harvesting the cells, a number of 120,000 cells required for the assay was adjusted in 1 ml PBS. The wells of a slide, provided by the manufacturer, were coated with agarose at 37 °C. After the agarose layer was formed by gelation, it was overcoated with 10 µl of cell suspension in 70 µl of agarose (1:8). Then, after repeated gelation of the agarose cell suspension, the cells were lysed for 1 h at 4 °C, with the provided lysis buffer. The slide was then pre-incubated in alkaline solution for 30 min. Subsequently, the gel electrophoresis was performed in alkaline solution as running buffer for 20 min at 18 V (1 V/cm). After immersing the slide twice in cold double-distilled H_2_O for 2 min, it was incubated in 70% cold ethanol for 5 min. Finally, the staining of the cells was performed using Vista Green DNA dye (1:10,000 in TE buffer) for 15 min. Analysis was performed by using the GFP filter (450–490 nm) of an Olympus IX83 microscope.

### TUNEL assay

To detect and quantify apoptosis-induced DNA damage, the DeadEnd™ fluorometric TUNEL assay kit (Promega Corporation, Madison, WI, USA) was used according to the manufacturer’s instructions. Here, 25,000 cells of melanoma cell line MEL-JUSO, SK-MEL-28, or MV3 were seeded on round 13-mm coverslips in a 12-well plate and treated with siCtrl or miR-101-3p mimic for 72 h. After washing the cells two times with PBS, they were fixed on the coverslips with 4% formaldehyde in PBS (pH 7.4) for 25 min at 4 °C. After removing the fixative solution, the cells were permeabilized with 0.2% Triton X-100 solution for 5 min and subsequently washed twice with PBS. To continue the treatments, coverslips were transferred to a light-shielded staining chamber. Cells were incubated in 100 µl of equilibration buffer for 10 min before incubation with 50 µl of rTdT buffer for at least 1 h at 37 °C. To stop the enzyme reaction, cells were incubated with 50 µl of 2 × SSC buffer for 15 min and thereupon washed three times with PBS. Finally, nuclear staining was performed using DAPI (1:10,000 in PBS) for 30 min before the coverslips were fixed on slides using Aqua-Poly/Mount (Polysciences). For staining analysis, an Olympus IX83 inverted microscope was used in combination with Olympus CellSens Dimension software (version 2.3, Olympus).

### Luciferase reporter gene assays

By cloning a part of the 3′-UTR region of LMNB1 mRNA (639 nt) (NM_005564.1) with the miR-101-3p response element (MRE, 5′-gtactgt-3′) into a pGL3 promoter firefly luciferase reporter vector (Promega Corporation), the direct influence of miR-101-3p on LMNB1 mRNA was determined. These were amplified by PCR from cDNA of melanoma cells using the Phusion High Fidelity DNA Polymerase Kit (ThermoFisher Scientific, Waltham, MA, USA) with the following primers including the additional bases CAG to cleave efficiently and the restriction site of XbaI (TCTAGA): FW: 5′-CAG TCT AGA AAG GCA GGC CAG ACT GTT AC-3′ and RV: 5′- CAG TCT AGA TAC ACC AAG ACG CAC AGT GG-3′. To exclude other regulations, a construct with a mutated binding site for miR-101-3p in 3′ UTR of LMNB1 was cloned, and following primers were used: 5′[Phos]-TTA ATA ACT GTG CAG CTG GAA GGG G-3′ and 5′[Phos]-GTT CAG TGT CAA TAA TTC ACA TCT TGC-3′. The final constructs were verified by sequencing. For analyses, 200,000 melanoma cells were first seeded in one well of a six-well plate and float-transfected with miR-101-3p mimic or siCtrl, respectively, using Lipofectamine RNAiMAX as described in Sect. 2.3 for approximately 24 h. After subsequent medium change, the obtained firefly luciferase reporter constructs (FLuc) and the empty vector as control (pGL3 promoter) were transfected with the Lipofectamine LTX transfection kit (Invitrogen, Thermo Fisher Scientific) for 24 h, as described elsewhere [42]. The Renilla reporter pRL-TK (RLuc; Promega Corporation, Madison, WI, USA) was transfected as control for normalization. The assay was performed using the Dual Luciferase^®^ Reporter Assay System from Promega. For the analysis, the FLuc signals were normalized to the corresponding RLuc signals by calculating the RLuc/FLuc ratio, considering the different transfection efficiencies. Luciferase assays were performed as described [43].

### miRNA sequencing bioinformatic sequence data analysis

The miRNA sequencing was performed as described elsewhere (GSE174334) [44].

### RNA-Seq library preparation, data preprocessing, and analysis

The total RNA sample isolation, library preparation and quality check, RNA sequencing, mapping of resulting paired-end reads, and the generation of raw and normalized counts was performed as previously published [27]. DESeq2 (v1.28.1) [45] was used for logarithmic transformation of the data, differential expression analysis, and calculation of adjusted *p*-values using the Benjamini–Hochberg method. Enrichment analysis were performed for significantly regulated genes (*p*_adj_ < 0.1) with EnrichR [46], for all expressed genes with GSEA (1000 × permutation, MSigDB v 2022, Signal2Noise metric, classical weighting), [47] and for putative miR-101-3p target genes with EnrichR html version using an intersection of all significantly downregulated genes in mimic transfected cells of the performed RNA-seq with direct and indirect miR-101-3p target genes from TarBase version 8.0 [48–50] and, in another analysis, from miRTarBase version 9.0 [51]. EnrichR html analysis was performed against a background of all identified genes of the RNA-seq, miRTargetLink 2.0, and GeneTrail 3.0 analysis [52] of the putative miR-101-3p target genes from the intersection of TarBase version 8.0 [48] annotated miR-101-3p target genes and significantly downregulated genes in mimic transfected cells [49, 50]. Enriched and overrepresented gene sets were classified as significant with a false discovery rate (FDR) or adjusted *p*-value (*p*_adj_) below 0.25. For data processing and visualization, the R packages “ggplot” and “dplyr” were used [53, 54].

### Statistical analysis

Statistical analysis was performed using the GraphPad Prism 9 software package (version 10.0.1; GraphPad Software Inc., San Diego, CA, USA). The results are shown as mean ± standard error of the mean (SEM). Comparisons between the groups were conducted using Student’s unpaired *t*-test, one-way analysis of variance (ANOVA) with subsequent Tukey’s multiple comparison test, or two-way ANOVA with subsequent Fisher’s least significant difference (LSD) multiple comparison test, respectively. Unless otherwise indicated, the number of independent experiments was at least *n* = 3. *p* ≤ 0.05 was considered statistically significant (*ns*, not significant).

## Results

### Loss of miR-101-3p is functionally relevant for melanoma progression

**Fig. 1:**
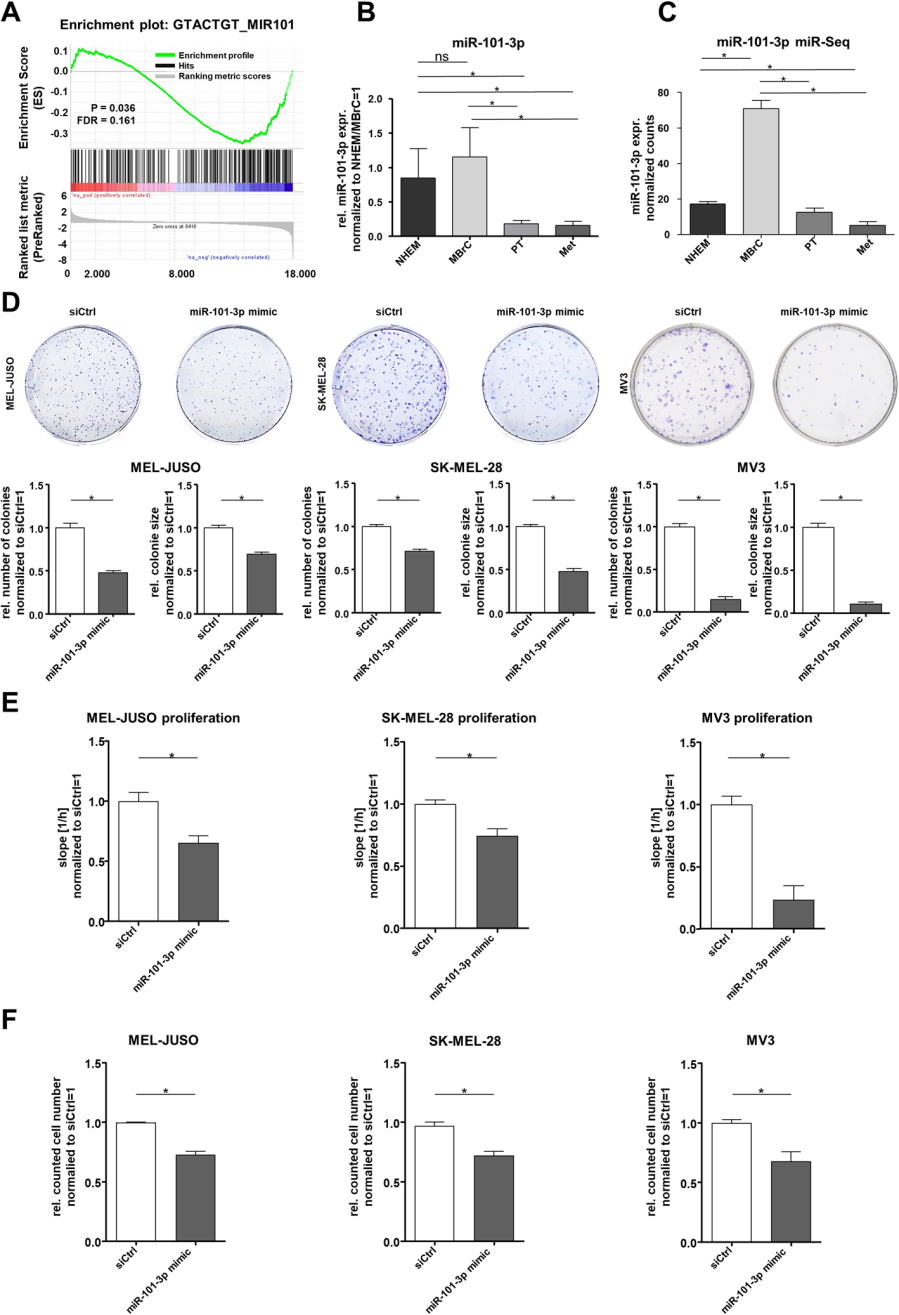
Loss of miR-101-3p supports the oncogenic properties of melanoma. **A** GSEA analysis: enrichment plot for miR-101-3p comparing a preranked gene list based on published data [29]. **B** Quantitative real-time PCR analysis of miRNA expression of miR-101-3p in NHEM, MBrC and primary tumor (PT) and metastasis (Met) melanoma cell lines (one-way ANOVA and subsequent Tukey’s multiple comparison test). **C** miRNA sequencing analysis (normalized counts) of different MM cell lines compared with NHEM and MBrCs (one-way ANOVA and subsequent Tukey’s multiple comparison test) [44]. **D** Clonogenic assay with MEL-JUSO, SK-MEL-28, and MV3 treated with miR-101-3p mimic (72 h) and respective siCtrl. Graphs show number of colonies and size of colonies. Representative images of colonies of MEL-JUSO, SK-MEL-28, and MV3 stained with Crystal Violet, treated with miR-101-3p mimic and siCtrl (Student’s *t*-test). **E** Real-time cell analysis in dependence on impedance measurement of MEL-JUSO, SK-MEL-28, and MV3 treated with miR-101-3p mimic (72 h) and respective siCtrl (Student’s *t*-test). **F** Counted cell number after transfection of MEL-JUSO, SK-MEL-28, and MV3 with miR-101-3p mimic (72 h) monitoring showed reduced proliferation compared with siCtrl (Student’s *t*-test). Bars represent mean ± SEM (*p ≤ 0.05, *ns* not significant)

We postulate that specific microRNAs, which are differentially expressed in malignant melanoma (MM) compared with melanocytes and MBrCs, are drivers of melanoma development and progression and stabilize the tumor phenotype. To investigate this hypothesis, we used miRNA expression analyses [18, 55, 56] and an in vitro model-based differential expression analysis [29, 30, 44]. With this bioinformatic analysis, we showed previously that many microRNAs have an almost equal expression level in NHEMs and MBrCs but are significantly differentially regulated in primary tumor- and metastasis-derived melanoma cell lines [44]. To find microRNAs, which act as drivers of melanoma development and progression, we bioinformatically analyzed a cDNA array, generated in our group, with GSEA using a provided ranked gene list (Additional file 2: Table S1) [29]. Premised on the literature, we have frequently found miR-101-3p to be often dysregulated in various tumor entities [24, 57–61]. Therefore, miR-101-3p was the most promising miRNA following our hypothesis based on the bioinformatic analysis (Fig. 1A). The enrichment plot of the GSEA analysis (green) using computational predicted miR-101-3p target genes [62, 63] showed a significant enrichment of downregulated miR-101-3p target genes (blue) in MM compared with NHEM and MBrC. For confirmation of differential expression of the newly identified microRNA, we performed quantitative real-time PCR (qRT-PCR), which showed significant loss of miR-101-3p in several melanoma cell lines compared with the expression in NHEM and MBrCs (Fig. 1B). Moreover, we analyzed the expression of miR-101-3p in our own miRNA-sequencing analysis (GSE174334) [44] in malignant melanoma cell lines compared with NHEM and MBrCs, which confirmed the results of the qRT-PCR (Fig. 1C). Focusing on miR-101-3p, we functionally characterized the effects of reexpression of this miRNA using in vitro assays in three melanoma cell lines after miR-101-3p mimic transfection (Fig. 1D, E). Reexpression after miR-101-3p mimic transfection was validated by qRT-PCR (Additional file 1: Fig. S1A). In clonogenic assays, miR-101-3p reexpression in the melanoma cell line MEL-JUSO, SK-MEL-28, and MV3 led to a reduced colony size and reduced number of colonies. This indicated that the miR-101-3p has an impact on clonogenicity and proliferative properties of melanoma. To further investigate this finding, we performed real-time cell analyses (RTCA) to monitor the proliferation rate of the cells, which revealed that the reexpression of miR-101-3p leads to a significant reduction of proliferation in the three melanoma cell lines (Fig. 1E, Additional file 1: Fig. S1B). The reduction in cell number (Fig. 1F) and concentration of total protein (Additional file 2: Fig. 1C) in miR-101-3p mimic-transfected melanoma cells compared with siCtrl underpinned the reduced proliferation rate. Additionally, we determined using RTCA and clonogenic assays as a proof of concept that the overexpression of miR-101-3p in NHEM has no effect on proliferation and clonogenicity, and also validated the overexpression of miR-101-3p in NHEM after miR-101-3p mimic transfection and the respective target genes EZH2 and LMNB1 by qRT-PCR (Additional file 2: Fig. 2A–D). In summary, loss of miR-101-3p supports the oncogenic properties of melanoma.

**Fig. 2:**
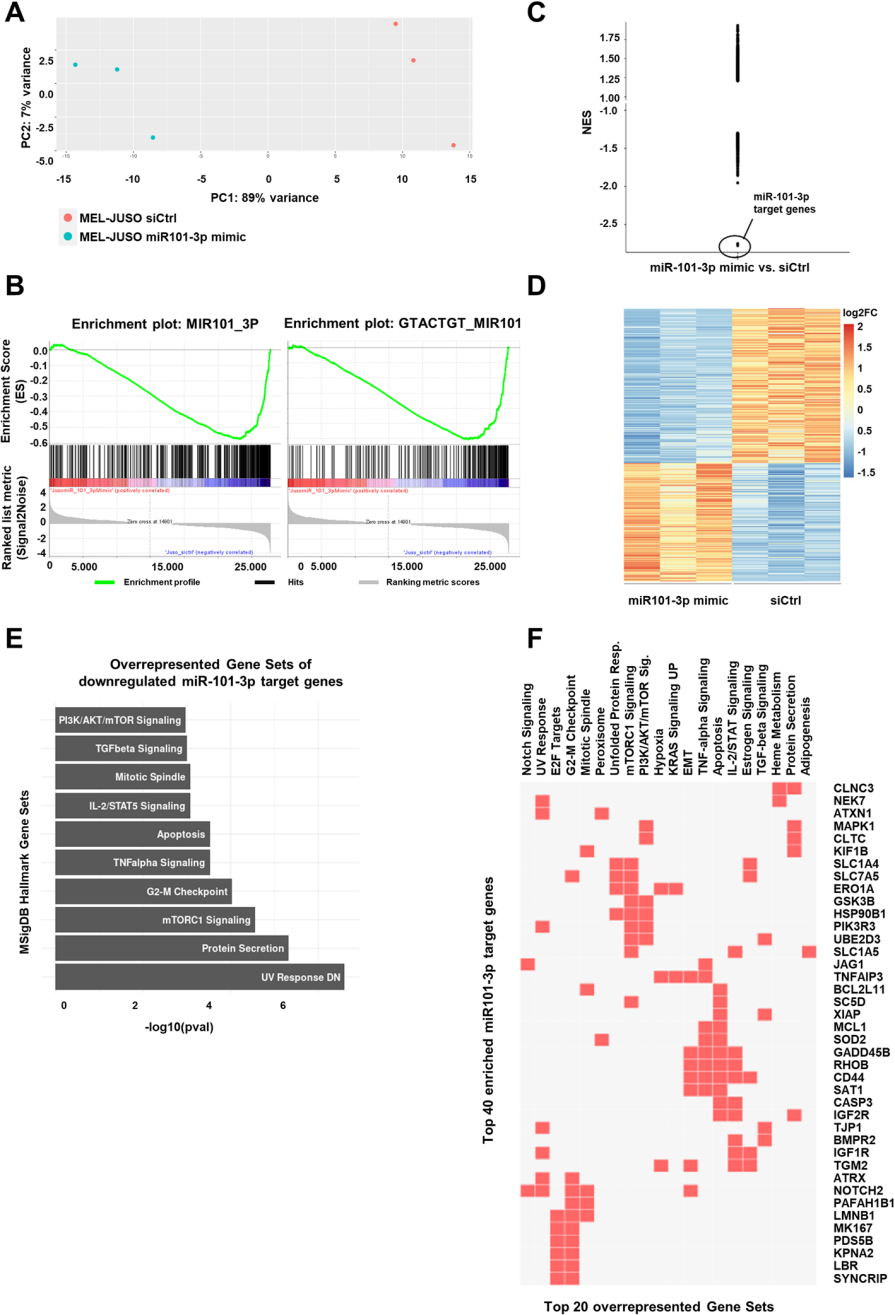
Predicted role of miR-101-3p in melanoma based on transcriptome analyses and bioinformatic analyses. **A** Principal component analysis of log-transformed normalized counts of the RNA-seq, showing clear differences in the gene expression of miR-101-3p mimic- and siCtrl-transfected cells. GSEA enrichment plot (**B**) and distribution of all significantly enriched miR-target gene sets. (**C**) Scatter plot of all significant normalized enrichment scores (NES with FDR < 0.25) showing the influence of miR-101-3p in regards of microRNA target genes in miR-101-3p mimic-transfected cells. **D** Heatmap of significantly up- (red) and downregulated (blue) miR-101-3p target genes found in miR101-3p mimic compared with siCtrl-transfected cells. **E** Top 10 overrepresented hallmark gene sets from MSigDB v22 of downregulated miR-101-3p target genes using EnrichR html version. **F** Overlap of 40 enriched miR101-3p target genes within the top 20 overrepresented gene sets determined by EnrichR analysis using clustergram [93]

### Predicted role of miR-101-3p in melanoma on the basis of transcriptome analyses

As shown in Fig. 1, the loss of miR-101-3p in tumor development is functionally relevant for melanoma progression. To define the molecular function and biological meaning of miR-101-3p expression in melanoma, RNA sequencing (RNA-seq) of miR-101-3p mimic-transfected primary melanoma cell line MEL-JUSO was performed (PRJNA841450). A principal component analysis of log-transformed normalized counts of the RNA sequencing clearly showed strong changes in the expression pattern in MEL-JUSO transfected with miR-101-3p mimic compared with siCtrl (Fig. 2A). These data were further analyzed by a differential gene expression analysis, EnrichR enrichment of significantly regulated genes and a gene set enrichment analysis (GSEA) of all expressed genes. First, we were interested in whether the reexpression of miR101-3p has the expected effects on the expression of putative miR-101-3p regulated target genes. Using the C3miR gene sets from MSigDB v22 of computational predicted miR-101-3p target genes and the normalized counts, these possible miR-101-3p target genes are highly and significantly enriched in siCtrl-transfected cells (Fig. 2B, Additional file 3: Table S2). Plotting of all normalized enrichment scores (NES) clearly exhibits an enrichment of those computational predicted miR-101-3p target genes in siCtrl-transfected cells by a wide margin to other miR-target gene sets (NES −2.78 and −2.75 for MIR101_3P and GTACTGT_MIR101 gene set, respectively; Fig. 2C). This showed the lack of computationally predicted miR-101-3p-mediated target gene regulation in siCtrl-transfected melanoma cells and validates the corresponding possible miR-101-3p target genes as the strongest downregulated ones of all predicted miR targets in miR-101-3p mimic-transfected cells. To receive a better overview of the up- and downregulated genes after miR-101-3p mimic transfection, we performed differential gene expression analysis. This analysis resulted in 2277 significantly upregulated and 2124 significantly downregulated genes in miR-101-3p mimic-transfected melanoma cells compared with siCtrl (*p*_adj_ < 0.1; Additional file 4: Table S3), which are also visualized in a volcano plot in Additional file 1: Fig. S3. To understand the changes in melanoma phenotype, we analyzed the defined biological function of significantly regulated genes after miR-101-3p mimic transfection with an EnrichR analysis using several different bioinformatical databases (genes with *p*_adj_ < 0.1, Additional files 2, 5: Fig. S4A, Table S4). Many different GO terms and pathways including apoptosis, p53 signaling, and RNA translation were found to be enriched by the reexpression of miR-101-3p, but there was no unambiguous change in the cellular phenotype observed by using only the significantly regulated genes.

Therefore, we performed a Gene Set Enrichment Analysis (GSEA, v4.3.2) [47, 64] with the normalized counts of all expressed genes found in RNA-Seq. Using hallmark gene sets from MSigDB, all the changes in expression resulted in 24 and 0 gene sets enriched significantly in miR-101-3p mimic- and siCtrl-transfected MEL-JUSO cells, respectively. Strikingly, epithelial–mesenchymal transition (EMT), apoptosis, DNA repair, and p53 pathway were among others enriched after miR-101-3p reexpression in melanoma cells (Table 1, Additional file 6: Table S5).

**Table 1:** Hallmark gene sets from MSigDB based on GSEA with normalized counts of all expressed genes found by RNA-Seq, showing significantly enriched pathways after miR-101-3p reexpression in MEL-JUSO compared with siCtrl

POS	NAME	SIZE	NES	FDR	POS	NAME	SIZE	NES	FDR
1	EPITHELIAL MESENCHYMAL TRANSITION	182	2.4	< 1.0 × 10^−4^	11	INTERFERON GAMMA RESPONSE	178	1.52	2.0 × 10^−2^
2	TNFA SIGNALING VIA NFKB	190	2.1	< 1.0 × 10^−4^	12	KRAS SIGNALING UP	162	1.49	2.4 × 10^−2^
3	INTERFERON ALPHA RESPONSE	90	1.9	6.1 × 10^−4^	13	P53 PATHWAY	181	1.48	2.5 × 10^−2^
4	IL6 JAK STAT3 SIGNALING	68	1.9	1.2 × 10^−3^	14	COAGULATION	111	1.47	2.7 × 10^−2^
5	APOPTOSIS	146	1.7	6.6 × 10^−3^	15	INFLAMMATORY RESPONSE	167	1.45	3.0 × 10^−2^
6	UNFOLDED PROTEIN RESPONSE	107	1.7	9.4 × 10^−3^	16	TGF BETA SIGNALING	53	1.33	8.7 × 10^−2^
7	HYPOXIA	182	1.6	9.5 × 10^−3^	17	ALLOGRAFT REJECTION	148	1.30	9.9 × 10^−2^
8	DNA REPAIR	147	1.6	1.8 × 10^−2^	18	COMPLEMENT	173	1.30	9.9 × 10^−2^
9	UV RESPONSE DN	135	1.5	2.0 × 10^−2^	19	PROTEIN SECRETION	94	1.29	9.8 × 10^−2^
10	ANGIOGENESIS	27	1.5	1.8 × 10^−2^	20	CHOLESTEROL HOMEOSTASIS	73	1.28	1.0 × 10^−1^

Next, we analyzed the expression of experimentally supported miR-101-3p target genes, which show differential expression in the RNA-Seq data, and their possible biological meaning. For this analysis, we used the TarBase version 8 database [65] with direct or indirect experimental evidence of microRNA targets. Here, we merged expressed genes of the RNA-seq with annotated miR-101-3p target genes from the TarBase version 8.0 database [65]. In general, TarBase version 8 annotated miR-101-3p target genes showed lower expression overall in mimic-transfected cells compared with siCtrl-transfected cells (Additional file 1: Fig. 4B). The expression of 2630 genes of 2827 annotated miR-101-3p targets was found in our RNA-seq, of which 1042 were significantly regulated (Fig. 2D). About 57% of those were downregulated in miR-101-3p mimic-transfected cells (Additional file 7: Table S6). To examine the biological meaning, we focused on these 589 identified significantly downregulated miR-101-3p target genes that were experimentally supported in mimic-transfected cells, because this kind of regulation is mainly expected after miR-101-3p reexpression. Overrepresentation analysis of significantly downregulated miR-101-3p target genes from TarBase version 8.0 was performed via EnrichR against all expressed genes of our RNA-seq data as background (https://maayanlab.cloud/EnrichR/enrich?dataset=35bfa59d07dcd979001c8b56bd71a91e). The 589 significantly downregulated miR-101-3p target genes (Additional file 7: Table S6) resulted here in similar MSigDB hallmark (v2020) gene sets as when using all differentially regulated genes (see above), namely, e.g., UV Response, G2-M Checkpoints, Apoptosis, and others (Fig. 2E, Additional files 8, 9: Tables S7 S8). Those enriched hallmark gene sets are clustering into four groups (Fig. 2F and Additional file 1: Fig. 4C), related to apoptosis, TNFalpha, TGF-beta, and IL2 signaling, related to PI3, protein secretion, and mTOR signaling, related to apoptosis/EMT and mitosis/E2F target genes. Additionally, another database was used, namely miRTarBase version 9.0 [51]. In total, 358 genes were found to be possible miR-101-3p target genes with either strong or weak experimental evidence, of which 127 were in common with significantly downregulated genes of the RNA-seq. Using EnrichR html against the same background as before, those possible miR-101-3p target genes were revealed to be relevant regarding G2-M Checkpoint and Mitotic spindle, apoptosis, and TNFalpha signaling as well as UV response (Additional file 10: Table S9, https://maayanlab.cloud/Enrichr/enrich?dataset=47b6d62c9028a6f97896b5101dd76c91). Similar results were achieved when using miRTargetLink 2.0 and embedded overrepresentation analysis via GeneTrail 3.0 (Additional file 11: Table S10) [52]. We additionally confirmed the genes ATRX, CASP3, and PARP to be significantly downregulated after miR-101-3p reexpression in MEL-JUSO, SK-MEL-28, and MV3 compared with siCtrl by qRT-PCR. These findings experimentally support that these genes are direct target genes of miR-101-3p (Additional file 1: Fig. S5A, B, C). Conclusively, the reexpression of miR-101-3p in MEL-JUSO resulted in a significant downregulation of genes that might play key roles in different biological processes such as apoptosis and cell cycle progression, leading to the validated functional effects.

### miR-101-3p affects nuclear processes in melanoma cells by targeting Lamin B1

**Fig. 3:**
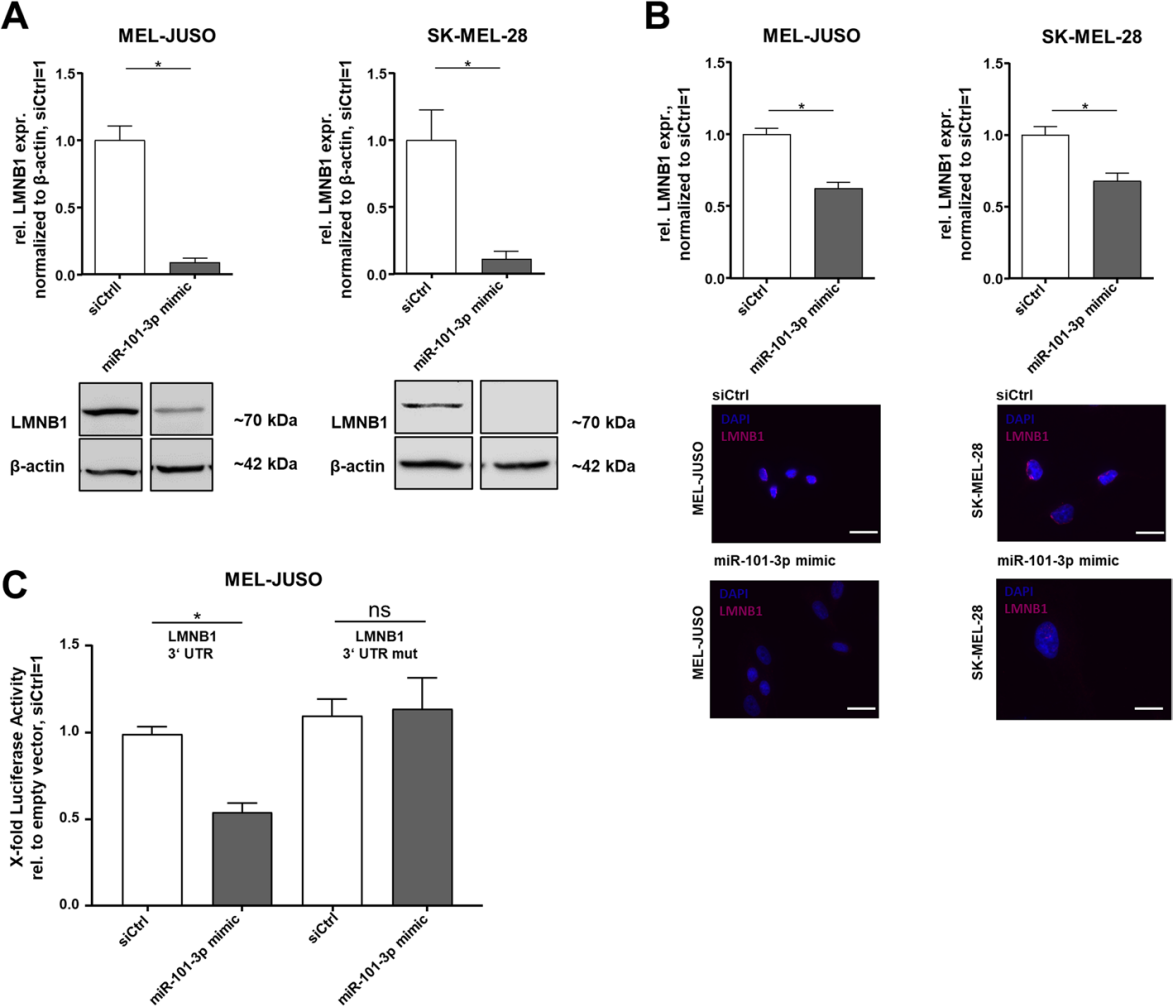
miR-101-3p directly targeted LMNB1. **A** Western Blot analysis of LMNB1 protein levels in MEL-JUSO and SK-MEL-28 transfected with miR-101-3p mimic and siCtrl, respectively (Student’s *t*-test). **B** Immunofluorescence staining of protein expression of LMNB1 in MEL-JUSO and SK-MEL-28 treated with miR-101-3p mimic (72 h) and respective siCtrl. Representative images of LMNB1 expression (red) in MEL-JUSO and SK-MEL-28 transfected with miR-101-3p mimic and siCtrl, respectively. Nuclear staining with DAPI (blue). Scale bars, 20 µm (Student’s *t*-test). **C** Luciferase assay of a pGL3prom construct with a miR-101-3p binding site for LMNB1 in the 3′-UTR and the corresponding mutagenesis constructs, revealing LMNB1 as a direct target of miR-101-3p (one-way ANOVA and subsequent Fisher’s LSD multiple comparison test). Bars show mean ± SEM (*p ≤ 0.05, *ns* not significant)

An interesting and predicted regulated target gene of miR-101-3p is the nuclear protein Lamin B1 (LMNB1), which plays a role in chromatin regulation and is a component of the nuclear skeleton [66]. Moreover, it is known to be involved in important nuclear processes [67–69]. Previously published data from our group revealed that LMNB1 influences the heterochromatin state and plays an important role in aging of melanoma cells [27]. Using Western Blot analyses, we determined a significantly reduced LMNB1 expression after reexpression of miR-101-3p in MEL-JUSO and SK-MEL-28 compared with transfected control cells (Fig. 3A). Moreover, this differential regulation was also validated by immunofluorescence staining of LMNB1 after miR-101-3p mimic transfection of MEL-JUSO and SK-MEL-28 (Fig. 3B). These results indicate that LMNB1 might be a direct target of miR-101-3p. For further evidence, we generated a luciferase reporter gene construct (LMNB1 3’UTR reporter) containing a conserved binding site for miR-101-3p. For exclusion of secondary effects, we also used a LMNB1 3′-UTR mut reporter containing a mutated binding site for miR-101-3p (Fig. 3C). The performed luciferase reporter gene assay clearly confirmed that LMNB1 is a direct target of miR-101-3p (Fig. 3C). Moreover, we have already demonstrated earlier that knockdown of LMNB1 leads to senescence induction and affects chromatin structure in malignant melanoma [27]. Conclusively, miR-101-3p directly regulates LMNB1 expression to stabilize nuclear processes in melanoma cells.

### miR-101-3p reexpression leads to apoptosis induction and affects the cell cycle

**Fig. 4:**
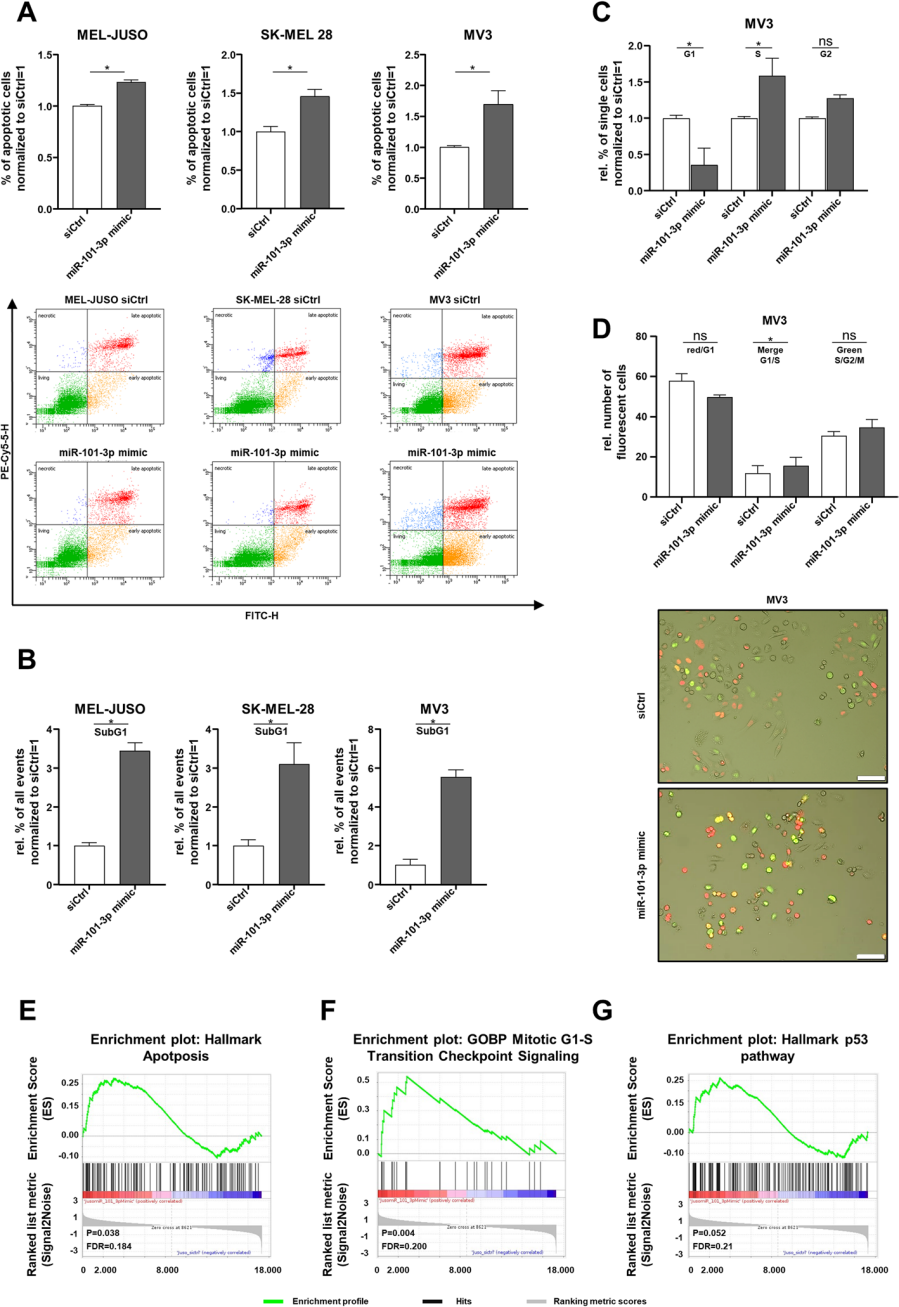
Reexpression of miR-101-3p and its influence on apoptosis and cell cycle. **A** Flow cytometry analysis for apoptotic cell detection in miR-101-3p mimic-transfected MEL-JUSO, SK-MEL-28, and MV3 (72 h) compared with siCtrl using Annexin V-FITC and PI staining (Student’s *t*-test). The pictures indicate one exemplary staining of MEL-JUSO, SK-MEL-28, and MV3. **B** Flow cytometry with the fluorescent dye propidium iodide for cell cycle staining of sub-G1 phase. MEL-JUSO, SK-MEL-28, and MV3 transfected with miR-101-3p mimic (72 h) and siCtrl, respectively (Student’s *t*-test). **C** Flow cytometry with the fluorescent dye propidium iodide for cell cycle staining of G1, S, and G2 phase. MV3 transfected with miR-101-3p mimic (72 h) and siCtrl, respectively (two-way ANOVA and subsequent Fisher’s LSD multiple comparison test). **D** Cell cycle analysis with the FUCCI reporter system in MV3 cells transfected for 72 h with miR-101-3p mimic and the respective siCtrl. Representative fluorescence staining with bright-field overlay of MV3 FUCCI miR-101-3p mimic- and siCtrl-transfected cells. Scale bars, 20 μm (two-way ANOVA and subsequent Fisher’s LSD multiple comparison test). **E**–**G** GSEA enrichment plots of the gene sets Hallmark Apoptosis, GOBP of Mitotic G1-S Transition Checkpoint Signaling, and Hallmark P53 Pathway (FDR < 25%), illustrating the profile of the running enrichment score (green) and positions of the enriched gene set and the rank-ordered list of genes differentially expressed in MEL-JUSO treated with miR-101-3p mimic and siCtrl, respectively. Genes upregulated in miR-101-3p mimic melanoma cells are shown on the left side of the graph in red, and downregulated ones on the right side in blue. Bars show mean ± SEM (*p ≤ 0.05, *ns* not significant)

Since LMNB1 has a crucial role for maintaining nuclear shape and mechanical integrity, we focused more on nuclear processes such as apoptosis. Moreover, the GSEA results implied the involvement of miR-101-3p in apoptotic processes as well. Therefore, we investigated miR-101-3p mimic-transfected cells by flow cytometry analysis for apoptosis. As shown in Fig. 4A, we determined a significant induction of apoptosis in all investigated cell lines after miR-101-3p mimic transfection compared with siCtrl. Analyzing the cell cycle with flow cytometry using the fluorescent dye propidium iodide (PI) showed that there are significantly more PI-positive cells in the SubG1 phase after miR-101-3p mimic transfection compared with siCtrl (Fig. 4B), which reinforces the finding of induction of apoptosis. An effect on the cell cycle after miR-101-3p mimic transfection was assumed according to the validated effect on reduced proliferation rate (Fig. 1E). Using PI staining, the effect on the whole cell cycle was analyzed in detail in the cell line MV3 after miR-101-3p mimic transfection, showing a significant delay in S/G2/M transition compared with siCtrl (Fig. 4C). Using the FUCCI reporter system in MV3, we investigated the cell cycle on the basis of different fluorescent states of the reporter by counting the respective cells with regards to their fluorescent dye. Conclusively, we also detected a significant delay in S/G2/M transition in MV3 FUCCI cells after miR-101-3p mimic transfection compared with siCtrl, respectively (Fig. 4D), which confirms the results of the flow cytometry analysis for cell cycle. However, in MEL-JUSO and SK-MEL-28, no significant effect was observed in S/G2/M transition (Additional file 1: Fig. S6A). The bioinformatical GSEA revealed a significant induction of apoptosis-associated genes after miR-101-3p reexpression in melanoma as well, which also underpins the experimental validation (Fig. 4E). Moreover, the GSEA results represented in Fig. 4F, G support the observed delay in S/G2/M transition because of the predicted upregulation of genes regarding Mitotic_G1_S_Transition_Checkpoint_Signaling and the upregulation of genes regarding the Hallmark_P53 pathway. This indicates that the loss of miR-101-3p in melanoma is necessary for a stable DNA replication. Conclusively, miR-101-3p reexpression affects the cell cycle progression and leads to apoptosis.

### Genomic instability was increased by miR-101-3p reexpression, resulting in cell death induction

**Fig. 5:**
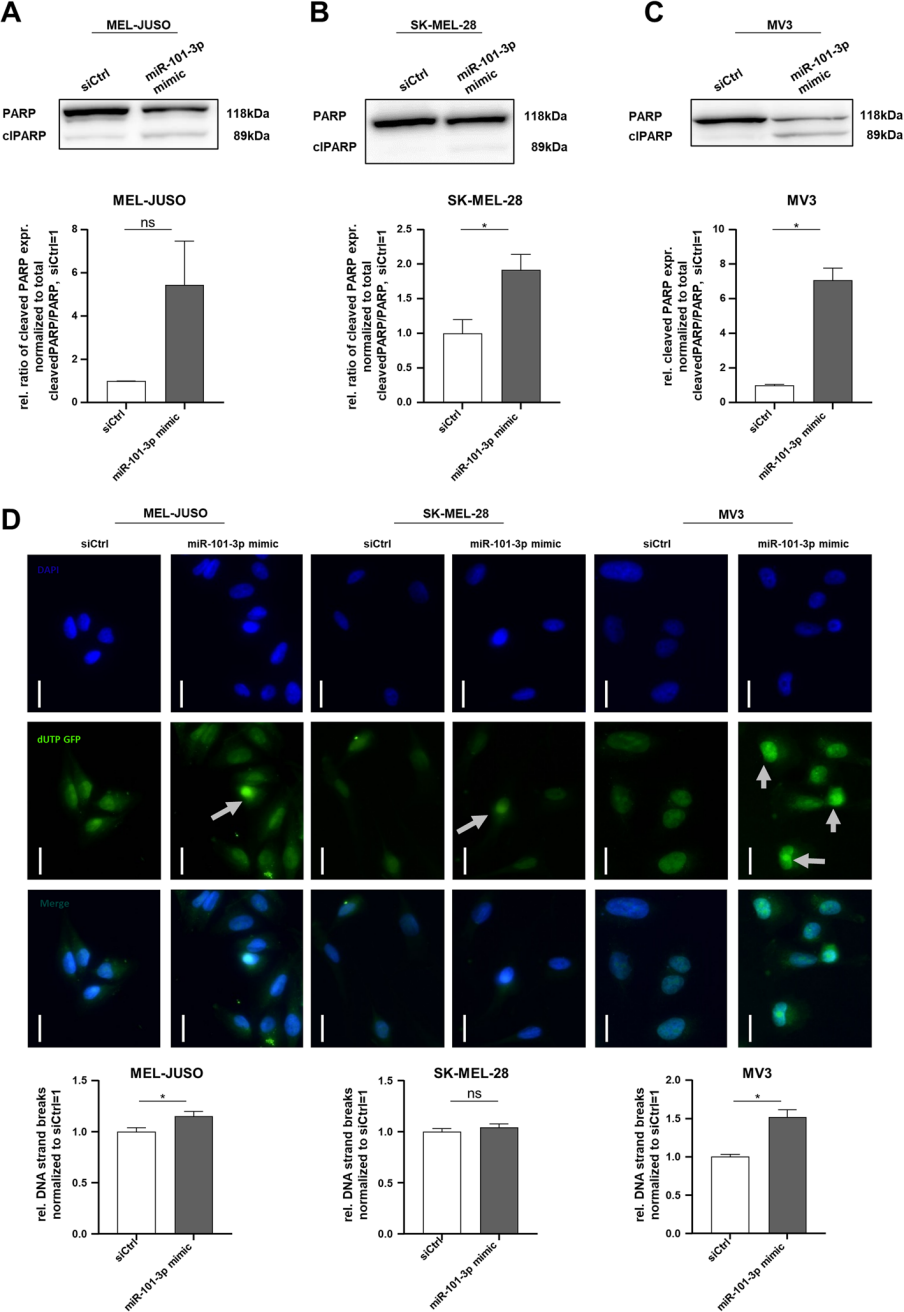
DNA damage induced apoptosis by PARP cleavage and TUNEL assay in miR-101-3p mimic- and siCtrl-treated cells. **A**–**C** Western blot analysis of full-length PARP and cleaved PARP following 72 h treatment with miR-101-3p mimic and siCtrl in MEL-JUSO, SK-MEL-28, and MV3 using the total of full-length PARP and cleaved PARP for calculating the ratio of cleaved PARP. Representative images of PARP western blots in MEL-JUSO, SK-MEL-28, and MV3 (Student’s *t*-test). **D** TUNEL assay for apoptosis detection by DNA strand breaks. Quantification and representative images of dUTP nick-end labeled DNA-strands (DAPI, GFP, Merge) of MEL-JUSO, SK-MEL-28, and MV3 transfected with miR-101-3p mimic (72 h) and siCtrl, respectively. Scale bars, 20 µm (Student’s *t*-test). Bars show mean ± SEM (*p ≤ 0.05, *ns* not significant)

We next focused on determining the functional apoptotic effects. First, we supported the induction of apoptosis by the expression of cleaved poly (ADP-ribose) polymerase 1 (PARP), which is increased in miR-101-3p mimic-transfected MEL-JUSO, SK-MEL-28, and MV3 cells (Fig. 5A–C, Additional file 1: Fig. S6B, C, D) compared with siCtrl-transfected cells. Despite this, we observed that the mRNA expression and protein expression of full-length PARP was significantly reduced in miR-101-3p mimic-transfected cells compared with siCtrl, respectively (Additional file 1: Figs. S5C, 6B, C, D). PARP is a molecule that is known to be relevant for DNA repair; therefore, this leads to the assumption that miR-101-3p reexpression might influence DNA repair mechanisms. According to PARP downregulation after miR-101-3p mimic reexpression, the DNA repair mechanisms might be reduced, leading to an accumulation of DNA strand breaks in the nucleus and increased genomic instability, which then results in cell death induction. To confirm and relate DNA damage to the validated apoptosis induction, a TUNEL assay in MEL-JUSO, SK-MEL-28, and MV3 transfected with miR-101-3p mimic and siCtrl, respectively, was performed (Fig. 5D). Although for SK-Mel-28 only a trend to increased DNA damages was detected, significantly more DNA strand breaks in MEL-JUSO and MV3 treated with miR-101-3p mimic compared with siCtrl were observed. In conclusion, miR-101-3p reexpression in melanoma cells leads to DNA damage, affecting DNA repair mechanisms and resulting in induction of apoptosis.

### Reexpression of miR-101-3p promotes genomic pressure in melanoma

**Fig. 6:**
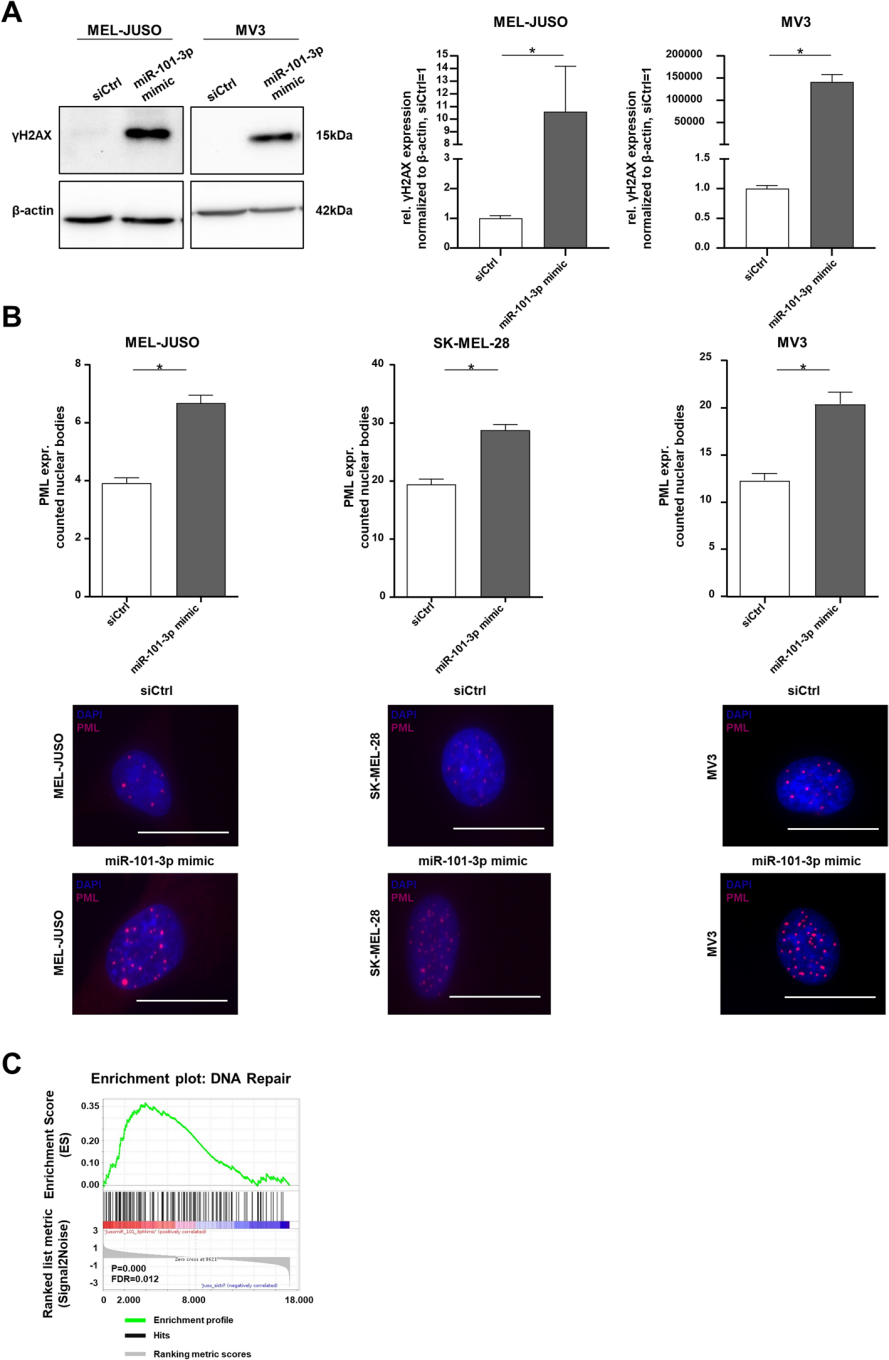
Reexpression of miR-101-3p and its influence on DNA damage and stress induction. **A** Western blot analysis of γH2AX following treatment 72 h with miR-101-3p mimic and siCtrl in MEL-JUSO and MV3 using β-actin as housekeeper (Student’s *t*-test). **B** Immunofluorescence staining of promyelocytic leukemia protein (PML) expression in MEL-JUSO, SK-MEL-28, and MV3 transfected with miR-101-3p mimic and siCtrl for 72 h as DNA damage detection (Student’s *t*-test). Representative images of PML nuclear bodies (red) and nuclear staining with DAPI (blue). Scale bars, 20 µm. **C** GSEA enrichment plot of Hallmark DNA_Repair (FDR < 25%), illustrating the profile of the running enrichment score (green) and positions of the enriched gene set and the rank-ordered list of genes differentially expressed in melanoma cells treated with miR-101-3p mimic and siCtrl, respectively. Genes upregulated in miR-101-3p mimic melanoma cells are shown on the left side of the graph in red, and downregulated ones on the right side in blue. Bars show mean ± SEM (*p ≤ 0.05, *ns* not significant)

As mentioned above, miR-101-3p seems to be involved in regulating cellular processes, leading to DNA damage and affecting DNA repair. This is supported by the hampered S/M/G2 transition, the respective reduced PARP expression, and the observed results from the TUNEL assay after miR-101-3p mimic transfection. For further validation, we used a broad spectrum of assays for DNA damage detection. Hence, the significantly increased phosphorylation of H2AX, a commonly used marker for DNA damage, reflecting DNA double strand breaks, was confirmed in MEL-JUSO and MV3 (Fig. 6A). The effect of miR-101-3p reexpression on DNA damage in MV3 was also supported by the comet assay (Additional file 1: Fig. S6E), a common assay for detection of DNA fragmentation, which is a hallmark of apoptosis. Furthermore, we detected significantly more nuclear bodies of promyelocytic leukemia protein (PML) in miR-101-3p mimic-transfected MEL-JUSO, SK-MEL-28, and MV3 compared with siCtrl cells (Fig. 6B). This proves not only DNA damage but also stress induction. Moreover, the bioinformatic prediction of the increased upregulation of genes involved in DNA repair mechanisms based on the GSEA analysis underpins the increase in DNA damage (Fig. 6C). We, therefore, speculate that the reexpression of miR-101-3p in melanoma leads to stress induction in cells, which increases the genomic pressure, leading to DNA single-strand breaks as well as DNA double-strand breaks resulting in apoptosis induction. Conclusively, miR-101-3p influences DNA-related processes and nuclear mechanisms, and its loss in melanoma provides the cells with genomic stability.

## Discussion

MicroRNAs are important regulator molecules of gene expression in normal and pathologic states such as cancer [70]. They are involved in various cellular processes and can either promote tumor progression or act as tumor suppressive [12]. In the present study, we focus on miR-101-3p in melanoma, whose role is still unknown. In NHEMs and MBrCs, miR-101-3p shows a high expression level, whereas it is lost in melanoma development. This suggests that the loss of miR-101-3p in melanoma plays a significant role in the manifestation of the tumor phenotype and the maintenance of a carcinogenic character in the cells. Therefore, miR-101-3p is not relevant for the transition between differentiation and dedifferentiation in the development of melanoblasts to NHEM but rather for the tumor onset from NHEM to primary melanoma. Since miR-101-3p remains lost even in metastatic melanoma cell lines, we have attributed it significance for further melanoma progression. The fact that miR-101-3p acts as tumor suppressive by regulating proliferation and cell death pathways according to posttranscriptional gene regulation supports our hypothesis.

There are prominent examples of miRNAs acting as tumor suppressive in melanoma, such as miR-196a [56], miR-193a-3p and -5p [71], miR-339-3p [72], miR-125b [19], miR-622 [18], and let-7 [55]. To date, the defined role of miR-101-3p in melanoma is, however, still unknown. On the basis of the revealed loss of miR-101-3p in melanoma, we examined functional effects by reexpressing miR-101-3p in the primary melanoma cell line MEL-JUSO and the metastatic cell lines SK-MEL-28 and MV3. Here, we demonstrated that the loss of miR-101-3p plays a crucial role for maintaining the oncogenic properties, such as clonogenicity and proliferation, of melanoma cells. Hence, we postulate that miR-101-3p is acting as tumor suppressive. Our results are supported by other studies in different cancer entities, which describe the tumor-suppressive role of miR-101-3p regarding important cellular processes. Here, miR-101-3p reduced proliferation and promoted apoptotic processes in breast cancer and non-small-cell lung cancer cells [73–76]. Additionally, the loss of miR-101-3p supports metastatic processes in breast cancer, glioblastoma, and ovarian cancer [60, 77, 78].

For melanoma, the sole published study on miR-101-3p suggests an inhibitory role in tumor progression. It indicates that miR-101-3p targets enhancer of zeste homolog 2 (EZH2) and microphthalmia-associated transcription factor (MITF), resulting in inhibition of proliferation and invasion [79]. These findings are in agreement with our own results. Conclusively, we wanted to define the functional and molecular role of miR-101-3p in melanoma in more detail using an unbiased method. Using a bioinformatic approach, we revealed that miR-101-3p reexpression is involved in DNA damage mechanisms by repressing genes involved in UV response and various genes resulting in apoptosis induction (Additional file 9: Table S8). On the basis of this finding, we confirmed that miR-101-3p reexpression leads to single DNA-strand breaks (sDSB) as well as double DNA-strand breaks (dDSB), as validated by significantly increased phosphorylation of H2AX, increased cleaved PARP, and reduced PARP expression, as well as positive TUNEL staining in melanoma cells. Additionally, it is known that the existence of improperly repaired or persistent dDSB may induce genomic instability [80]. Interestingly, the upregulation of γH2AX has already been linked to the induction of apoptosis induced by genomic instability in cancer [81]. This is also supported by the conducted EnrichR analysis, which revealed that X-linked inhibitor of apoptosis (XIAP) is targeted by miR-101-3p. XIAP is reported to be overexpressed in melanoma cells [82]. XIAP is the only IAP family member that can inhibit both the intrinsic and extrinsic apoptotic pathways by binding to caspase-9, caspase-7, and caspase-3, respectively [83, 84]. Conclusively, miR-101-3p reexpression affects mechanisms of the intrinsic and/or the extrinsic apoptotic pathways by targeting XIAP, leading to apoptosis induction on its own. Moreover, we confirmed CASP3 to be significantly downregulated after miR-101-3p reexpression compared with siCtrl, in accordance with the determined apoptosis induction. A study by Feng et al. revealed apoptosis‑promoting properties of miR‑3074‑5p in murine preosteoblast cells according to the downregulation of XIAP and CASP3, which underpins our findings for miR-101-3p in melanoma [85]. Furthermore, after reexpression of miR-101-3p, we revealed an upregulation of the DNA damage marker PML. The EnrichR analyses confirmed this by revealing that miR-101-3p targets the transcriptional regulator ATRX, which is described to localize with PML nuclear bodies [86]. The impact of ATRX on DNA damage is further supported by other studies. Gulve et al. linked a knockdown of ATRX in glioblastoma cell lines with ALT-like features to depletion of Histon H3 and to an upregulation of γH2AX [87]. The transcriptional regulator ATRX is known to contribute to heterochromatin, limits homologous recombination, and affects alternative lengthening of telomers (ALT) [88, 89]. Moreover, ATRX is described to interact with EZH2 [90], which has already been confirmed as a direct target of miR-101-3p in melanoma. Consequently, we showed bioinformatically that miR-101-3p targets ATRX and EZH2, which disturbs gene transcription at the chromatin level, giving the fact that both genes are involved in histone modulation [91]. In this context, we newly identified the nuclear structure protein LMNB1 as a direct target of miR-101-3p. In a previous study, we showed that LMNB1 is upregulated in several melanoma cell lines [27]. The downregulation of LMNB1 leads to changes in the nuclear structure by influencing the heterochromatin structure [27]. As described in this study, LMNB1 is targeted by miR-101-3p and the reexpression of miR-101-3p in MV3 leads to a problem in S/G2/M transition. A study by Camps et al. determined that LMNB1 is involved in maintaining chromatin condensation in the interphase nuclei and silencing LMNB1 leads to a prolonged S-phase in colorectal cancer [92]. The bioinformatic EnrichR analysis based on our RNA-seq data of MEL-JUSO cells showed that miR-101-3p affects the G2–M checkpoint by targeting LMNB1. Conclusively, miR-101-3p reexpression leads to a prolonged S-phase by targeting LMNB1, which affects genomic integrity. With these data, we determined that the reexpression of miR-101-3p influences important nuclear processes, leading to genomic pressure, which can be associated with the experimentally determined DNA damage.

Finally, our findings show that the reexpression of miR-101-3p in different melanoma cell lines leads to a strong intervention in DNA repair pathways, resulting in DNA damage, which induces apoptosis. Consequently, the loss of miR-101-3p, as an early event in melanoma, leads to the stabilization of nuclear processes affecting replication. Therefore, the expression of important proteins such as EZH2, ATRX, and LMNB1 can be maintained or adapted to keep and stabilize the increased proliferation rate of melanoma cells. Moreover, owing to the loss of miR-101-3p, melanoma prevents apoptosis by XIAP upregulation. As a result, the reexpression of miR-101-3p in melanoma cells leads to increased genomic instability, which results in apoptosis induction. In summary, understanding the role of miR-101-3p provides new insights into melanoma progression by regulating gene expression of several genes involved in DNA repair and DNA damage processes and apoptotic pathways influencing genomic integrity.

## Conclusions

In melanoma, the mechanism of DNA replication is unstable owing to the increased proliferation rate of melanoma cells. This genomic instability is an important process for melanoma cells to adapt to the increased bioenergetic demand. The loss of miR-101-3p in the melanoma cell lines MEL-JUSO, SK-MEL-28, and MV3 stabilizes this process owing to upregulation of target genes of miR-101-3p, which are involved in DNA replication, chromatin stabilization, and Histon modifications. Additionally, this stabilization also leads to cell death prevention, which maintains the increased bioenergetic demand. Conclusively, miR-101-3p plays an important role in acting as tumor suppressive by regulating the proliferation and cell death pathways according to posttranscriptional gene regulation.

### Supplementary Information

**Additional file 1. Figure S1A.** Quantitative real-time PCR analysis of miRNA expression of miR-101-3p in MELJUSO,SK-MEL-28 and MV3 after miR-101-3p mimic transfection compared to siCtrl (72 h) (Student’s t-test). **Figure S1A.** Quantitative real-time PCR analysis of miRNA expression of miR-101-3p in MELJUSO, SK-MEL-28 and MV3 after miR-101-3p mimic transfection compared to siCtrl (72 h) (Student’s t-test). **Figure S1B.** Proliferation Curves of real time cell analysis in dependency of impedance measurement of MEL-JUSO, SKMEL-28 and MV3 treated with miR-101-3p Mimic (72 h) and respective siCtrl (Student’s t-test). **Figure S1C.** Total protein lysate after RIPA of MEL-JUSO, SK-MEL-28 and MV3 transfected with miR-101-3p mimic and siCtrl (72 h) (Student’s t-test). Bars represent the means ± SEM (* = p ≤ 0.05, ns = not significant). **Figure S2A.** Clonogenic assay with NHEM treated with miR-101-3p mimic (18 h) and respective siCtrl (n=1). Representative images of NHEM stained with crystal violet, treated with miR-101-3p mimic and siCtrl. Scale bars equal 100 μm. **Figure S2B.** Proliferation Curves of real time cell analysis in dependency of impedance measurement of NHEM treated with miR-101-3p Mimic (18 h) and respective siCtrl (n=1,) (Student’s t-test). **Figures S2C.** Quantitative real-time PCR analysis of miRNA expression of miR-101-3p in NHEM after miR-101-3p mimic transfection compared to siCtrl (18 h, n=1). **Figures S2D.** Quantitative real-time PCR analysis of RNA expression of EZH2 and LMNB1 in NHEM after miR-101-3p mimic transfection compared to siCtrl (18 h, n=1). **Figure S3. **Volcano Plot of differentially expressed genes in miR-101-3p mimic transfected cells compared to siCtrl-transfected cells analyzed by RNA-Seq. Significantly (p-value < 0.1) and strongly (log2 FoldChange > 1.5 or < −1.5 respectively) upregulated genes are marked in red and downregulated genes in blue. **Figure S4A.** EnrichR analysis of significant regulated genes after miR-101-3p transfection by using different bioinformatical databases: KEGG, WikiPathway, GO Biological Processes (genes with padj < 0.1). **Figure S4B.** Distribution of log(norm counts) of the expressed genes in miR-101-3p mimic and siCtrl, determining that miR-101-3p target genes show lower expression overall in mimic transfected cells compared to siCtrl. **Figure S4C.** Enriched hallmark gene sets which cluster in 4 clusters based on the overrepresentation analysis of significantly downregulated miR-101-3p target genes via EnrichR against expressed genes of the RNA-Seq. **Figure S5A,B,C.** qRT-PCR analysis of potential target genes ATRX, CASP3 and PARP determined by EnrichR analysis using clustergram (Overlap of 40 enriched miR101-3p target genes within the top 20 overrepresented gene sets) (Student’s t-test). Bars represent the means ± SEM (* = p ≤ 0.05, ns = not significant). **Figure S6A.** Flow cytometry with the fluorescent dye propidium iodide for cell cycle staining of G1, S and G2 phase. MEL-JUSO and SK-MEL-28 transfected with miR-101-3p mimic (72 h) and siCtrl respectively (two-way ANOVA and subsequent Fisher’s LSD multiple comparison test). **Figure S6B,C,D.** Western blot analysis of full-length PARP and cleaved PARP following treatment 72 h with miR-101-3p mimic and siCtrl in MELJUSO, SK-MEL-28 and MV3 using β-actinashousekeeper. Representative images of PARP Western blots in MELJUSO, SK-MEL-28 and MV3 (Student’s t-test). **Figure S6E.** Representative Comet-Assay images from fluorescence microscopy at 10x magnification showing the fragmented DNA migration from the nucleoid body which forms a comet tail in MV3 transfected with miR-101-3p mimic (72h) and siCtrl. The cytostatic etoposide was used as positive control respectively. Bars represent the means ± SEM (* = p ≤ 0.05, ns = not significant).

**Additional file 2.** SupplTab1_C3mir_GSEA.

**Additional file 3.** SupplTab2_allmirtargets_GSEA.

**Additional file 4.** SupplTab3_DEG.

**Additional file 5.** SupplTab4_EnrichR.

**Additional file 6.** SupplTab5_GSEA_Hallmark_mimic.

**Additional file 7.** SupplTab6_miR101_targetgenes_down_padj.

**Additional file 8.** SupplTab7_EnrichR_hallmarkv20_miR101_down.

**Additional file 9.** SupplTab8_miR_padj_dwn_hallmarks.

**Additional file 10.** SupplTab9_tarbase_mirtarbase_enrichresults.

**Additional file 11.** SupplTab10_miRTargetLink_GeneTrail.

## Data Availability

RNA sequencing data will be deposited in the SRA Submission Portal database prior to publication.

## Abbreviations

ATRX: ATP-dependent helicase ATRX
CASP3: Caspase 3
dDSB: Double DNA-strand breaks
DMEM: Dulbecco’s modified Eagle’s medium
EZH2: Enhancer of zeste homolog 2
FBS: Fetal bovine serum
FLuc: Firefly luciferase reporter constructs
FUCCI: Fluorescent ubiquitination-based cell cycle indicator
GSEA: Gene Set Enrichment Analysis
H2AX: H2A histone family member X
KRAS: Kirsten rat sarcoma
LMNB1: Lamin B1
MBrC: Melanoblast-related cells
miRNAs: MicroRNAs
MITF: Melanocyte inducing transcription factor
MM: Malignant melanoma
NHEM: Normal human epidermal melanocytes
ns: Not significant
PARP: Poly(ADP-ribose)-polymerase
PBS: Phosphate buffer saline
PML: Promyelocytic leukemia protein
RIPA: Radioimmunoprecipitation assay
RPMI: Roswell Park Memorial Institute
RTCA: Real-time cell analysis
RT: Reverse transcriptase
SEM: Standard error of the mean
sDSB: Single DNA-strand breaks
TUNEL: TdT-mediated dUTP-biotin nick end labeling
XIAP: X-linked inhibitor of apoptosis
