# Supplementary material for: Loss of miR-101-3p in melanoma stabilizes genomic integrity, leading to cell death prevention
Source: Cell Mol Biol Lett. 2024 Mar 2;29:29. doi: 10.1186/s11658-024-00552-2 (PMC10909299; doi:10.1186/s11658-024-00552-2)
Supplement: Supplementary file 1 — Additional file 1. Figure S1A. Quantitative real-time PCR analysis of miRNA expression of miR-101-3p in MELJUSO,SK-MEL-28 and MV3 after miR-101-3p mimic transfection compared to siCtrl (72 h) (Student’s t-test). Figure S1A. Quantitative real-time PCR analysis of miRNA expression of miR-101-3p in MELJUSO, SK-MEL-28 and MV3 after miR-101-3p mimic transfection compared to siCtrl (72 h) (Student’s t-test). Figure S1B. Proliferation Curves of real time cell analysis in dependency of impedance measurement of MEL-JUSO, SKMEL-28 and MV3 treated with miR-101-3p Mimic (72 h) and respective siCtrl (Student’s t-test). Figure S1C. Total protein lysate after RIPA of MEL-JUSO, SK-MEL-28 and MV3 transfected with miR-101-3p mimic and siCtrl (72 h) (Student’s t-test). Bars represent the means ± SEM (* = p ≤ 0.05, ns = not significant). Figure S2A. Clonogenic assay with NHEM treated with miR-101-3p mimic (18 h) and respective siCtrl (n=1). Representative images of NHEM stained with crystal violet, treated with miR-101-3p mimic and siCtrl. Scale bars equal 100 μm. Figure S2B. Proliferation Curves of real time cell analysis in dependency of impedance measurement of NHEM treated with miR-101-3p Mimic (18 h) and respective siCtrl (n=1,) (Student’s t-test). Figures S2C. Quantitative real-time PCR analysis of miRNA expression of miR-101-3p in NHEM after miR-101-3p mimic transfection compared to siCtrl (18 h, n=1). Figures S2D. Quantitative real-time PCR analysis of RNA expression of EZH2 and LMNB1 in NHEM after miR-101-3p mimic transfection compared to siCtrl (18 h, n=1). Figure S3. Volcano Plot of differentially expressed genes in miR-101-3p mimic transfected cells compared to siCtrl-transfected cells analyzed by RNA-Seq. Significantly (p-value < 0.1) and strongly (log2 FoldChange > 1.5 or < −1.5 respectively) upregulated genes are marked in red and downregulated genes in blue. Figure S4A. EnrichR analysis of significant regulated genes after miR-101-3p transfection by using different bioi [file 11658_2024_552_MOESM1_ESM.pdf]

SupplFig1

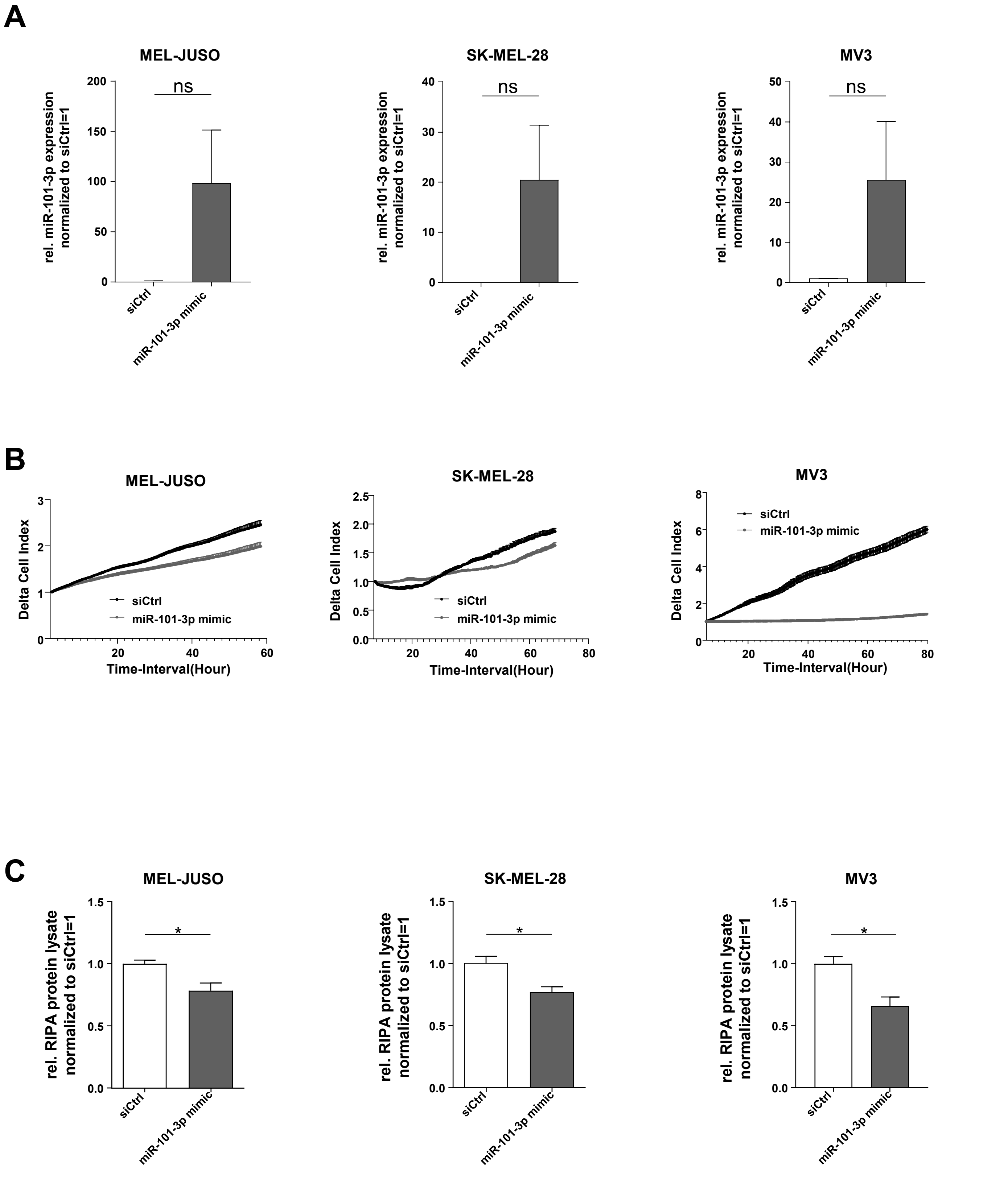

SupplFigure 1: (**A**) Quantitative real-time PCR analysis of miRNA expression of miR-101-3p in MEL-JUSO, SK-MEL-28 and MV3 after miR-101-3p mimic transfection compared to siCtrl (72 h) (Student's *t*-test). (**B**) Proliferation Curves of real time cell analysis in dependency of impedance measurement of MEL-JUSO, SK-MEL-28 and MV3 treated with miR-101-3p Mimic (72 h) and respective siCtrl (Student's *t*-test). (**C**) Total protein lysate after RIPA of MEL-JUSO, SK-MEL-28 and MV3 transfected with miR-101-3p mimic and siCtrl (72 h) (Student's *t*-test). Bars represent the means ± SEM (* = p ≤ 0.05, ns = not significant).

SupplFig2

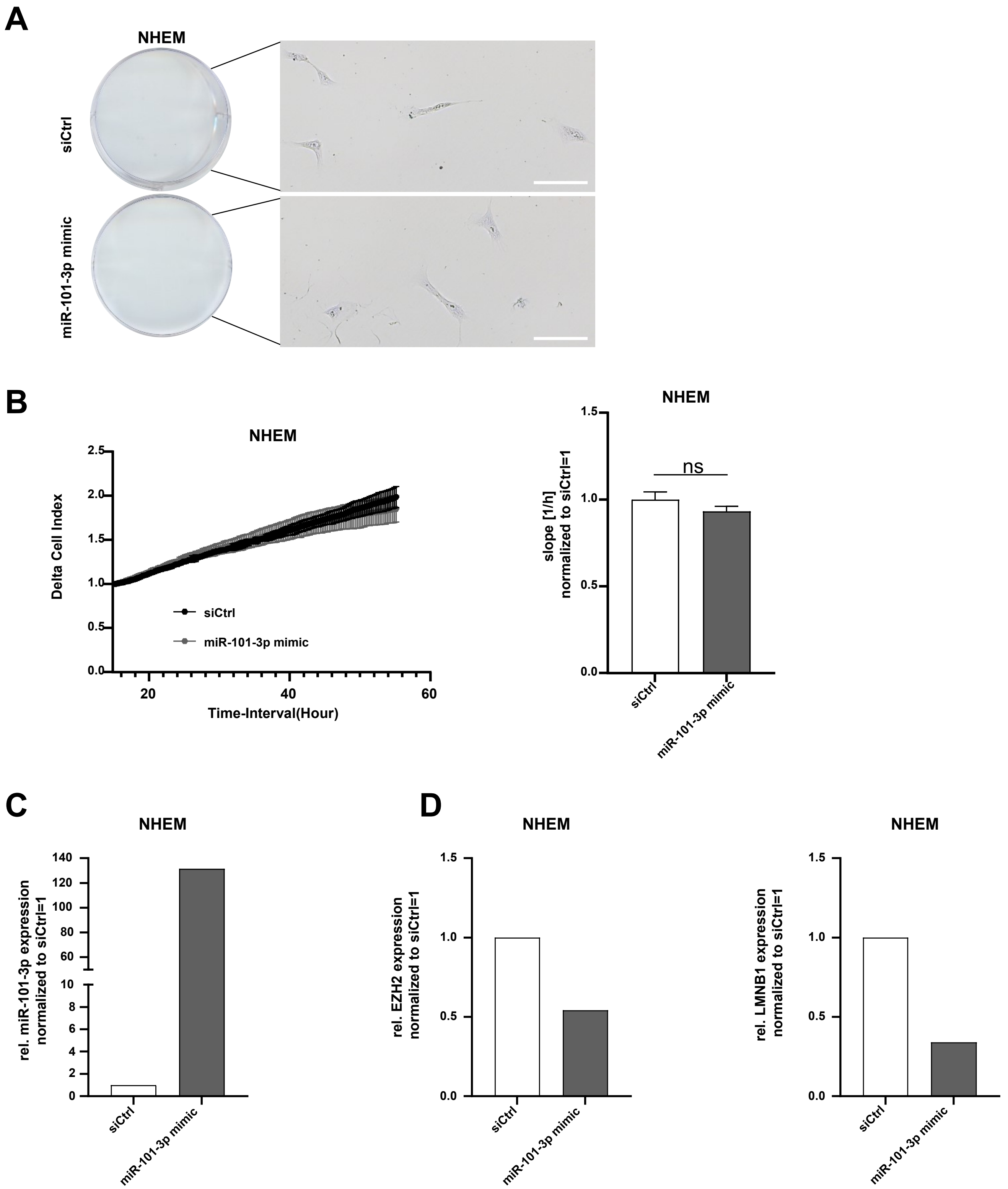

SupplFigure 2: (**A**) Clonogenic assay with NHEM treated with miR-101-3p mimic (18 h) and respective siCtrl (n=1). Representative images of NHEM stained with crystal violet, treated with miR-101-3p mimic and siCtrl. Scale bars equal 100 µm. (**B**) Proliferation Curves of real time cell analysis in dependency of impedance measurement of NHEM treated with miR-101-3p Mimic (18 h) and respective siCtrl (n=1,) (Student's *t*-test). (**C**) Quantitative real-time PCR analysis of miRNA expression of miR-101-3p in NHEM after miR-101-3p mimic transfection compared to siCtrl (18 h, n=1). (**D**) Quantitative real-time PCR analysis of RNA expression of EZH2 and LMNB1 in NHEM after miR-101-3p mimic transfection compared to siCtrl (18 h, n=1).

SupplFig3

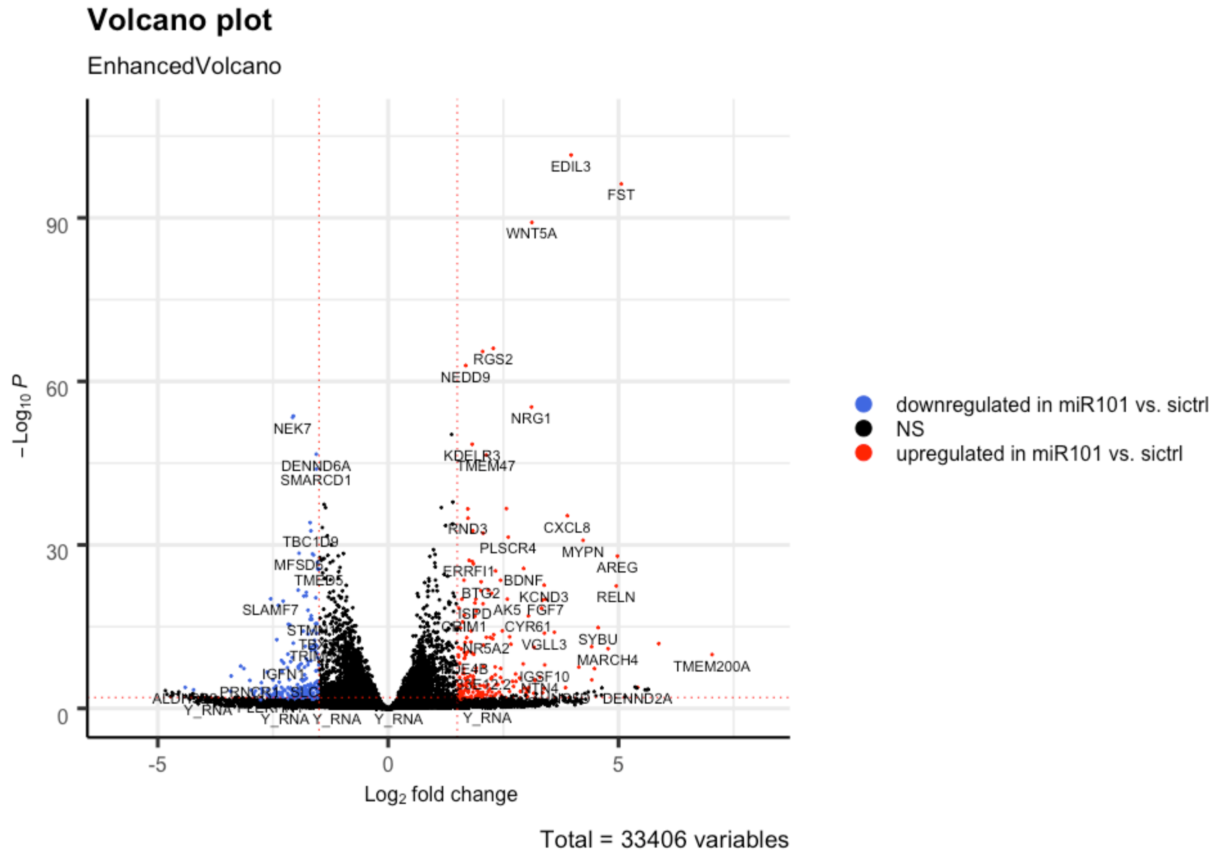

SupplFigure 3: Volcano Plot of differentially expressed genes in miR-101-3p mimic transfected cells compared to siCtrl-transfected cells analyzed by RNA-Seq. Significantly (p-value < 0.1) and strongly (log2 FoldChange > 1.5 or < -1.5 respectively) upregulated genes are marked in red and downregulated genes in blue.

SupplFig4

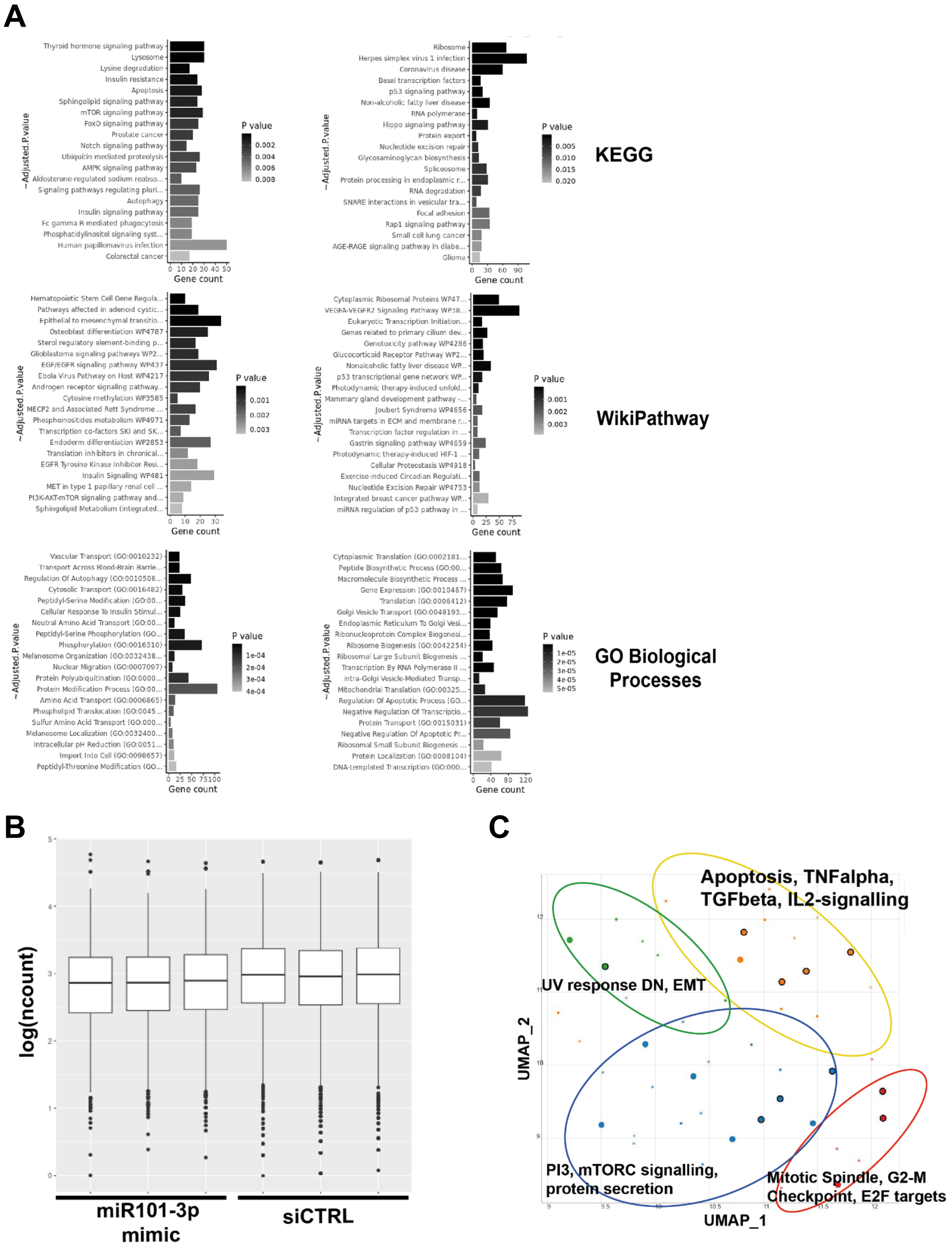

SupplFigure 4: (**A**) EnrichR analysis of significant regulated genes after miR-101-3p transfection by using different bioinformatical databases: KEGG, WikiPathway, GO Biological Processes (genes with padj < 0.1). (**B**) Distribution of log(norm counts) of the expressed genes in miR-101-3p mimic and siCtrl, determining that miR-101-3p target genes show lower expression overall in mimic transfected cells compared to siCtrl. (**C**) Enriched hallmark gene sets which cluster in 4 clusters based on the overrepresentation analysis of significantly downregulated miR-101-3p target genes via EnrichR against expressed genes of the RNA-Seq.

SupplFig5

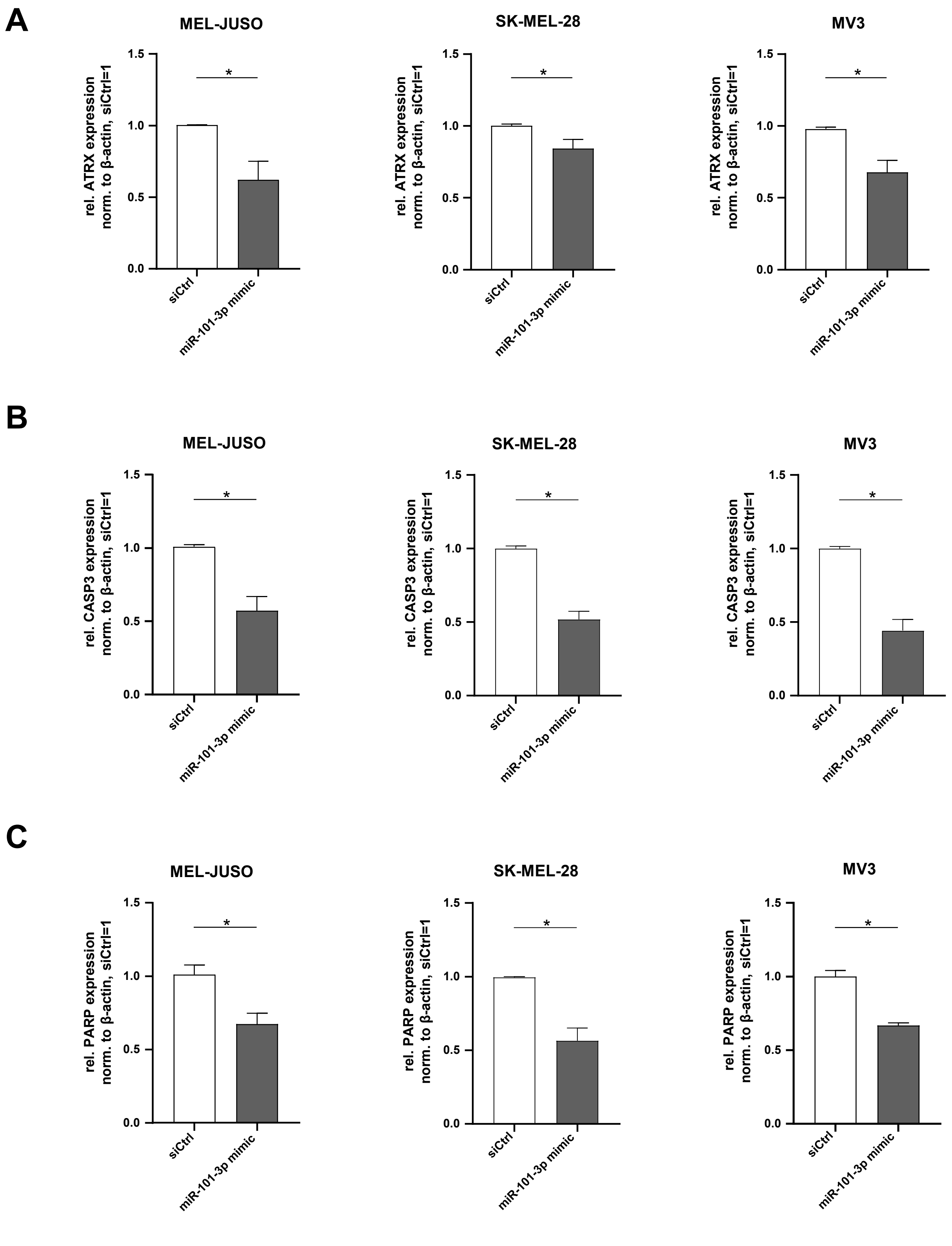

SupplFigure 5: (**A**, **B**, **C**) qRT-PCR analysis of potential target genes ATRX, CASP3 and PARP determined by EnrichR analysis using clustergram (Overlap of 40 enriched miR101-3p target genes within the top 20 overrepresented gene sets) (Student's *t*-test). Bars represent the means ± SEM (* = p ≤ 0.05, ns = not significant).

SupplFig6

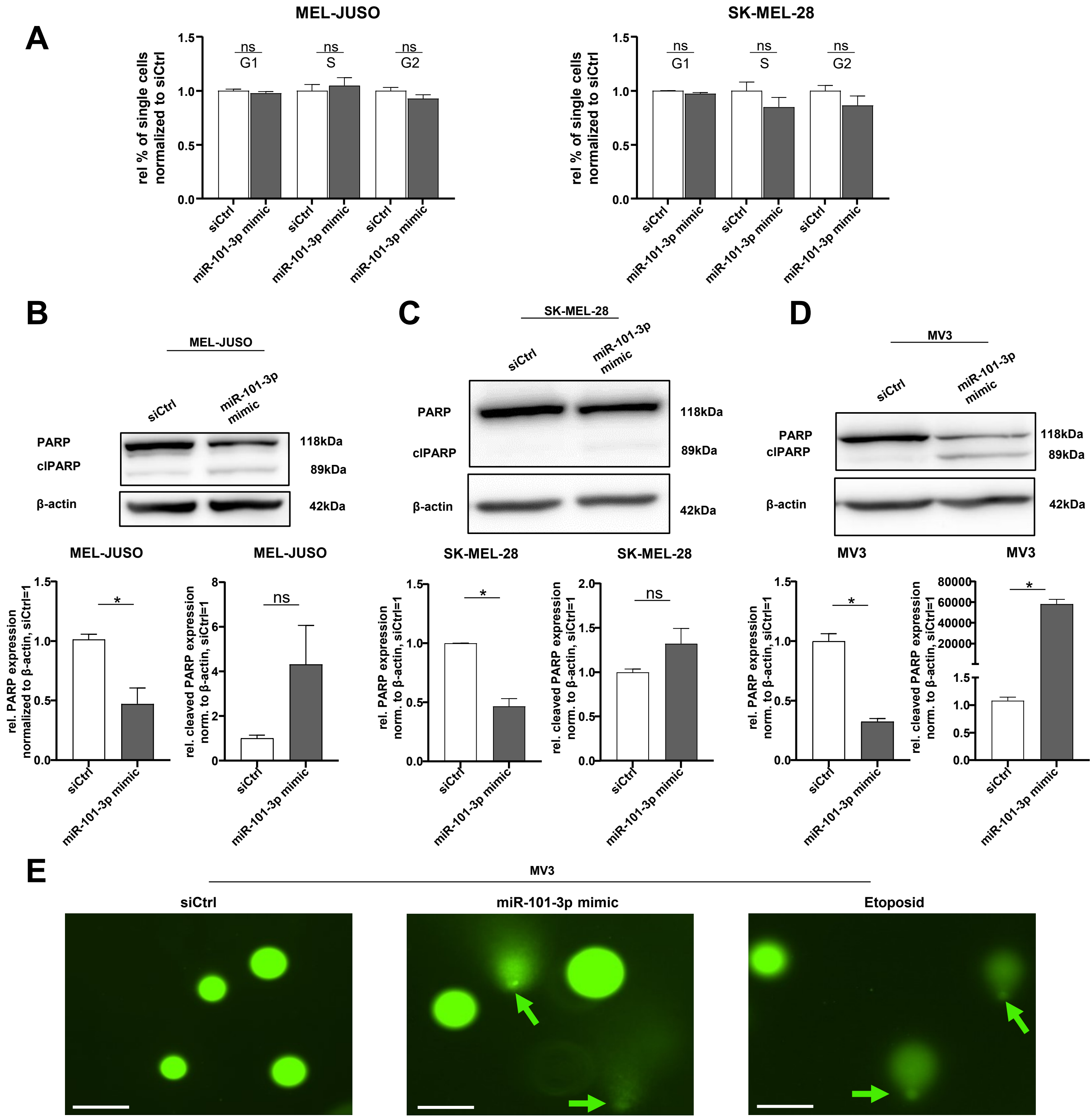

SupplFigure 6: (**A**) Flow cytometry with the fluorescent dye propidium iodide for cell cycle staining of G1, S and G2 phase. MEL-JUSO and SK-MEL-28 transfected with miR-101-3p mimic (72 h) and siCtrl respectively (two-way ANOVA and subsequent Fisher's LSD multiple comparison test). (**B, C, D**) Western blot analysis of full-length PARP and cleaved PARP following treatment 72 h with miR-101-3p mimic and siCtrl in MEL-JUSO,SK-MEL-28 and MV3 using β- actin as housekeeper. Representative images of PARP Western blots in MEL-JUSO, SK-MEL-28 and MV3 (Student's *t*-test). (**E**) Representative Comet-Assay images from fluorescence microscopy at 10x magnification showing the fragmented DNA migration from the nucleoid body which forms a comet tail in MV3 transfected with miR-101-3p mimic (72h) and siCtrl. The cytostatic etoposide was used as positive control respectively. Bars represent the means ± SEM (* = p ≤ 0.05, ns = not significant).
